# Molecular cloning and the expression profile of two calnexin genes – *CNX1* and *CNX2* – during pollen development and pollen tube growth in *Petunia*

**DOI:** 10.1186/s12870-025-07186-2

**Published:** 2025-10-23

**Authors:** Anna Suwińska, Piotr Wasąg, Marta Lenartowska, Jarosław Tyburski, Robert Lenartowski

**Affiliations:** 1 Department of Cellular and Molecular Biology, Faculty of Biological and Veterinary Sciences, Nicolaus Copernicus University in Toruń, Toruń, Poland; 2 Centre for Modern Interdisciplinary Technologies, Nicolaus Copernicus University in Toruń, Toruń, Poland; 3 Department of Plant Physiology and Biotechnology, Faculty of Biological and Veterinary Sciences, Nicolaus Copernicus University in Toruń, Toruń, Poland

**Keywords:** Calnexin, FISH, Gene cloning, Gene expression, Immunocytochemistry, Microsporogenesis, *Petunia hybrida*, Pollen tube

## Abstract

**Background:**

Calnexin (CNX) is a crucial chaperone of the endoplasmic reticulum (ER) that participates in the folding and quality control of glycoproteins. In plants, CNX contributes to multiple physiological processes, including growth, development, and adaptation to abiotic stresses. Nevertheless, its specific function in male gametophyte development and pollen tube growth remains poorly understood. In this work, we report for the first time the molecular cloning of two *Petunia hybrida CNX* homologs, *PhCNX1* and *PhCNX2*, examine their expression profiles during pollen development, germination, and tube elongation, and discuss their potential functional involvement in these processes.

**Results:**

We successfully cloned and characterized full-length cDNAs of *PhCNX1* and *PhCNX2*, which encode proteins containing conserved motifs typical of the CNX/CRT family. Through a combination of qRT-PCR, western blotting, fluorescence in situ hybridization (FISH), and immunocytochemistry, we show that both genes are expressed in *Petunia* anthers, germinating pollen grains, and elongating tubes. During male gametophyte development, *PhCNX1* shows peak expression at the microspore stage, whereas *PhCNX2* reaches its highest transcript levels in mature pollen grains. Notably, in dry pollen, both genes exhibit a marked decrease in transcript abundance. FISH analysis indicates that *PhCNX* mRNAs are detected in both somatic and germline tissues but are predominantly localized to the cytoplasm of tapetal cells from the microsporocyte to microspore stages. Moreover, western blot analysis reveals a progressive increase in CNX protein levels throughout anther development and its accumulation in dry pollen. Immunocytochemical staining confirms CNX localization in all anther cell types, with notable enrichment in tapetal cells and the cytoplasm of pollen grains prior to anther dehiscence; this is further supported by immunogold labeling indicating CNX localization within the ER. In germinating pollen and elongating tubes, *PhCNX* transcripts accumulate in the cytoplasm near the apertures and along the tube (except the clear zone), whereas CNX protein is concentrated in the ER-dense subapical region.

**Conclusion:**

Our findings reveal that *PhCNX1* and *PhCNX2* are dynamically regulated throughout the course of pollen development within the anther, as well as during pollen germination and tube growth. This spatiotemporal expression pattern supports the notion that CNX functions as a molecular chaperone facilitating the high levels of protein synthesis essential for proper male gametophyte maturation and the polarized growth of the pollen tube.

**Supplementary Information:**

The online version contains supplementary material available at 10.1186/s12870-025-07186-2.

## Introduction

**Fig. 1:**
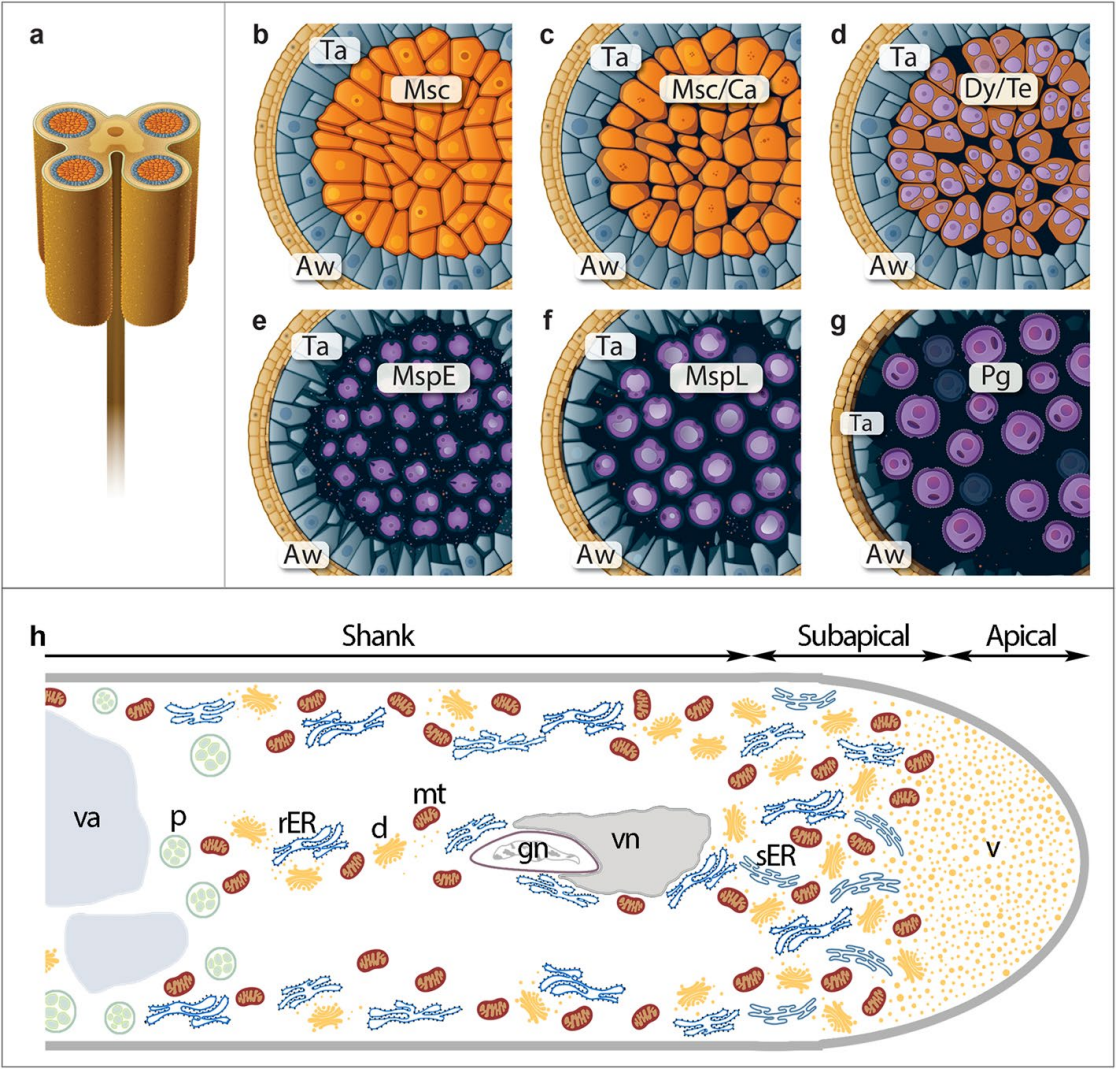
Scheme of microsporogenesis in angiosperms (**a**-**g**). *Cartoons* show cross sections of *Petunia* anther (**a**) during the next stages of its development including: microsporocyte (*Msc*) stage (**b**), callose (*Msc*/*Ca*) stage (**c**), dyad/tetrad (*Dy*/*Te*) stage (**d**), early microspore (*MspE*) stage (**e**), late microspore (*MspL*) stage (**f**), and pollen grain (*Pg*) stage (**g**). Diagram of the polar organization of angiosperm pollen tube cytoplasm (**h**). The cytoplasm exhibits three distinct zones: the apical zone (clear zone), packed with secretory and endocytic vesicles for tube tip growth; the subapical zone rich in metabolically active organelles (sER, rER, mitochondria, and Golgi stacks); and the shank zone, divided into the distal shank (housing the male germ unit (MGU), amyloplasts, vacuoles, rER, and some mitochondria and dictyosomes) and the proximal shank (dominated by large vacuoles and callose plugs). *Aw* anther wall, *d* dictyosome, *gn* generative nucleus, *mt* mitochondrium, *p* plastid, *rER* rough endoplasmic reticulum, *sER* smooth endoplasmic reticulum, *Ta* tapetum, *vn* vegetative nucleus, *v* vesicles, *va* vacuole

In flowering plants, pollen grain develops in the anther and germinates into a pollen tube that delivers immobile sperm cells to the embryo sac for double fertilization. Pollen formation is accompanied by tightly controlled events during microsporogenesis, involving both sporophytic and gametophytic tissues of the anther (Fig. 1a). Microsporogenesis (Fig. 1b-f) begins with the differentiation of diploid microsporocytes (Fig. 1b-c). These cells undergo meiosis to form dyads and then tetrads of haploid microspores surrounded by a callosic wall (Fig. 1d). Enzymatic degradation of this cell wall by callase, secreted by the nutritive tissue tapetum, releases young microspores (Fig. 1e). The microspores grow, become vacuolated (Fig. 1f), and finally undergo an asymmetric mitotic division giving two different cells (Fig. 1g). The smaller generative cell divides to produce two sperm cells, while the larger and highly active vegetative cell accumulates a bulk of mRNAs, proteins, lipids, and polysaccharides to form a pollen tube [1]. Pollen tubes can be cultivated in vitro, serving as an excellent research model for studying tip-growing plant cells. As the tube elongates, it becomes polarized, leading to the formation of three cytoplasmic zones: the apical, subapical and shank zones, which differ in both structure and function (Fig. 1h). The tip growth of the pollen tube is driven by the secretion of Golgi-derived vesicles, which are transported to the apical plasma membrane via actin-dependent cytoplasmic streaming [2]. To maintain a rapid growth, a high rate of protein synthesis, as well as other components of the cytoskeleton, cell membranes, and cell wall is strictly required. On the other hand, the formation of the multilayered pollen cell wall (sporoderm), which begins at the tetrad stage of microsporogenesis, also requires a high secretory activity of the microspores and tapetal cells. The biogenesis of the secretory proteins occurs in the ER [3, 4]. Due to its functions, the ER contains several chaperone proteins that prevent the aggregation of immature glycoproteins and control their proper folding. Molecular chaperones, such as calnexin (CNX) and its paralog calreticulin (CRT), play a key role in these processes.

CNX is highly conserved among plants, fungi, and animals [5, 6]. Unlike the soluble protein CRT, CNX functions as a type I integral membrane chaperone of the ER. Its structure includes domains typical of the CNX/CRT family, including a large luminal domain, a transmembrane helix, and a short C-terminal cytoplasmic tail with the RKPRRE motif, which serves as an ER retention signal [7, 8]. The amino acid sequences of CNX in plants and animals contain conserved regions in the middle parts and highly diverse regions at the N- and C-termini [5]. Along with CRT and additional ER proteins, CNX participates in the CNX/CRT cycle, which promotes the correct folding and oligomerization of newly synthesized glycoproteins prior to their export from the ER [9, 10]. CNX has also been implicated in Ca^2+^ level regulation [7, 11, 12], endocytosis [13], phagocytosis [14], cell–cell adhesion [15], and cell sensitivity to apoptosis [16–18]. The *CNX/CRT* superfamily, which includes *CNX* and *CRT* genes, represents a multigene family characterized by evolutionary relationships. Phylogenetic analyses indicate that *CNX* and *CRT* share a common ancestry, having originated from early ancestral gene duplications [19]. To date, CNX has been characterized in multiple plant species, revealing that some species possess multiple *CNX* genes, while others contain only a single copy [5, 20–27]. Studies on the expression of *CNXs* have revealed that these genes are present in a variety of plant tissues [22, 24]. They have been detected in leaves [5, 27], roots [23, 28], hypocotyls [23], cotyledons [29], seeds [24] and buds [24, 26], as well as in floral structures, including sepals, stigmas, stamens, and pollen grains [25]. Plant CNX is thought to play a role in responses to abiotic stress, vegetative growth, and development [24, 26, 29]. Given the extensive protein synthesis is required during pollen development, pollen germination, and pollen tube elongation, CNX likely serves as a molecular chaperone involved in protein folding and quality control in the ER during these processes. Indeed, in 2017, Vu et al. [29] showed that the proteins of the CNX/CRT cycle are essential for the vegetative growth in *Arabidopsis*, suggesting a critical role for CNX1 in pollen viability. However, the expression profiles of diverse CNXs during microsporogenesis and pollen tube growth remain unexplored. To address this research gap, we cloned and characterized the full-length cDNAs sequences of *PhCNX1* and *PhCNX2* genes and analyzed their expression patterns during the anther development, pollen germination, and pollen tube elongation. In our research we used model plant *Petunia*, which produces two-celled pollen grains. In contrast, *Arabidopsis* produces three-celled pollen grains, which is not typical for dicotyledonous plants. In the context of our previous studies on the expression profile and role of CRT in microsporogenesis and pollen tube formation in *Petunia* [30–33] and results provided by other authors, this work provides new results for the comparative analysis of the expression and function of CRT and CNX in key reproductive events of dicotyledonous plants.

## Methods

### Plant material and pollen tubes cultures

The plant material was obtained from commercial cultivars of *Petunia hybrida* (pistils, anthers, and pollen) or *Arabidopsis thaliana* (whole plant) grown at room temperature (Department of Cellular and Molecular Biology, Faculty of Biological and Veterinary Sciences, Nicolaus Copernicus University in Toruń, Poland). The whole pistils of *Petunia* were dissected from flowers 6 h after pollination. The anthers of *Petunia* were dissected at various stages of microsporogenesis, including microsporocytes, dyads/tetrads, microspores, and pollen grains. Collected pistils, anthers, dry pollen, and whole *Arabidopsis* plants were frozen in liquid nitrogen and stored at − 80 °C until RNA or protein isolation. To identify the specific stages of pollen development, freshly dissected anthers were crushed on a microscope slide, stained with 0.1% (*w*/*v*) aniline blue, and observed under a fluorescence microscope (Nikon Eclipse 80i). To analyze the histological features of the anther at each developmental stage, semi-thin sections of the fixed and embedded anthers were stained with 0.1% (*w*/*v*) methylene blue and examined using light microscopy (Nikon Eclipse 80i). For light microscopy, FISH, immunocytochemistry, and electron microscopy, dissected anthers were fixed, dehydrated in ethanol, and embedded according to the protocols described below. The cultures of *Petunia* pollen tubes were obtained as previously described [34]. Briefly, freshly collected mature pollen was germinated in liquid culture medium containing 0.2% sucrose, 0.05% Ca(NO_3_)_2_, 0.01% MgSO_4_, 0.01% H_3_BO_4_, 0.01% KNO_3_, 15% polyethylene glycol 4000, and 0.4% 2-(N-morpholino)ethanesulfonic acid (pH 6.0) at 30 °C for about 2 h. Germinated pollen and growing pollen tubes were prepared for FISH and immunocytochemistry as described below. All experiments were repeated at least three times using biological replicates collected during different growing seasons.

### Extraction of total RNA and cDNA synthesis

Plant material (100 mg of pistils or anthers) was ground into a powder in liquid nitrogen. Total RNA was isolated using RNA Extracol® (EURx) and treated with DNase I, RNase-free (Thermo Fisher Scientific) according to the manufacturer's protocols. The samples were then extracted with one volume of an acid-phenol:chloroform:isoamyl alcohol mixture (125:24:1; Merck), followed by one volume of chloroform. The extracted RNAs were precipitated with isopropanol, pelleted at 15,000 × g for 15 min (4 °C), and washed with ice-cold 70% ethanol. The concentration and purity of RNA were assessed using a NanoDrop One spectrophotometer (Thermo Fisher Scientific), and the quality of samples was inspected on a 1% agarose gel in 1 × TBE buffer.

To obtain the first-strand cDNA, 5 μg of total RNA was reverse transcribed using FIREScript® Enzyme Mix, 10 × RT Reaction Premix, and oligo(dT)/random primers (Solis BioDyne OÜ), following the manufacturer's instructions. Subsequently, all cDNA probes were purified with High Pure Product Purification Kit (Roche) according to the manufacturer’s protocol.

### Random amplification of cDNA ends (RACE)

RACE was carried out as described before [35]. In brief, for 3’ and 5’-RACE, gene-specific primers were designed based on two partial *CNX* cDNA sequences of *Petunia axilliaris* (Peaxi162Scf00314g00733.1 and Peaxi162Scf00207g00250.1) available in the Sol Genomics Network database. To obtain the missing parts of the *PhCNX1* and *PhCNX2* sequences, 5’-RACE was carried out using the FirstChoice RLM-RACE kit (Thermo Fisher Scientific) following the manufacturer’s protocol. Total RNA isolated from *Petunia hybrida* pistils was modified following the manufacturer’s protocol and then reverse transcribed in the presence of random decamers, the M-MLV enzyme and RNase inhibitor at 42 °C for 1 h. A 1-μl aliquot of the reverse transcription reaction was used in the outer 5’-RACE PCR in the presence of the 5’-RACE outer primer, 5’-RACE gene-specific outer primer (Table 1), and Platinum™ SuperFi™ DNA Polymerase. PCR cycles were as follows: 95 °C for 90 s, followed by 35 cycles of 95 °C for 30 s, 61 °C or 56 °C for 30 s (for 5’ *PhCNX1* or *PhCNX2* sequence, respectively), and 72 °C for 30 s, with a final extension step at 72 °C for 4 min. A 1-μl aliquot of the outer 5’-RACE PCR mixture served as the template in the inner 5’-RACE PCR using the 5’-RACE inner primer and 5’-RACE gene-specific inner primer (Table 1). PCR cycles were as described above.

**Table 1:** Primers used in RACE, RT-PCR, qRT-PCR, and sqRT-PCR

RACE	*CNX1*	5′-RACE GSP outer primer	5’ACCACAGTCCCCTCTTTGAGAT3’
		5′-RACE GSP inner primer	5’ATCATGTCCCTCGCTCTTCTC3’
	*CNX2*	5′-RACE GSP outer primer	5’GGGACGGAGGAATTTAAGATA3’
		5′-RACE GSP inner primer	5’GATAAGCACCACCACATTCAAGT3’
	*CNX1*	3′-RACE GSP outer primer	5’CTCGAGAAAGCTGAGAAGCAG3’
		3′-RACE GSP inner primer	5’CGTCGTCTCAGTCATAGTTGTT3’
	*CNX2*	3′-RACE GSP outer primer	5’ATCTTCTTGAAAAGGCCGAGAG3’
		3′-RACE GSP inner primer	5’TGTCGTCTCCACTGTTTTCTTG3’
RT-PCR	*PhCNX1*	Forward and reverse primers	5’TCTCACTCTCTTTTTATCTCTAAGTTCTAG3’ 5’CGTTCAAAATCTATGATATACAAAGGATACTC3’
	*PhCNX2*	Forward and reverse primers	5’ CCGCAAAAATTTCAACTCCTATTTCT3’ 5’GTTATGATAGAAAAGTATACCTTTGTATATATCGTC3’
qRT-PCR and sqRT-PCR	*18 rRNA*	Forward and reverse primers	5’GACTCAACACGGGGAAACTTAC3’ 5’CAGAACATCTAAGGGCATCACA3’
	*PhCNX1*	Forward and reverse primers	5’ATATGGGAAAAGGCACTAGCAA3’ 5’GCGATACATGCCTGTTTTGAA3’
	*PhCNX2*	Forward and reverse primers	5’GCTCATCTTTGGAGGAAAGAAA3’ 5’ATTGGTCCCTTTGGAATCTCTA3’

3’-RACE was carried out using the FirstChoice RLM-RACE kit (Thermo Fisher Scientific), according to the manufacturer’s protocol. Reverse transcription was performed using the M-MLV enzyme and 3’ RACE Adapter in the presence of an RNase inhibitor. A 1-μl aliquot of the reaction was then used in the outer 3’-RACE PCR with the 3’ RACE Outer Primer, 3’ RACE gene-specific outer primer (Table 1), and Platinum™ SuperFi™ DNA Polymerase. PCR cycles were as follows: 95 °C for 90 s, followed by 35 cycles of 95 °C for 30 s, 65 °C for 60 s, 72 °C for 60 s, with a final extension step at 72 °C for 4 min. A 1-μl aliquot of the outer 3’-RACE PCR mixture served as the template in the inner 3’-RACE PCR using 3’-RACE Inner Primer and 3’-RACE gene-specific inner primer (Table 1). PCR cycles were as described above.

### Cloning of the full-length cDNA sequences and molecular probe synthesis for PhCNX1 and PhCNX2 genes

To obtain the full-length *PhCNX1* or *PhCNX2* cDNA sequence, a 1-μl aliquot of the reverse transcribed total RNA from *Petunia* pistils was used as the template for PCR amplification with Platinum™ SuperFi™ DNA Polymerase (Thermo Fisher Scientific) and gene-specific primers (Table 1). PCR cycles were as follows: 95 °C for 90 s, followed by 35 cycles of 95 °C for 60 s, 56 °C or 60 °C for 90 s (for *PhCNX1* or *PhCNX2* sequences, respectively), and 72 °C for 90 s, with a final extension step at 72 °C for 4 min. The RT-PCR products were visually inspected on a 1% agarose gel in 1 × TAE buffer.

The full-length *PhCNX1* and *PhCNX2* cDNAs obtained by RT-PCR were recombined into the pCR4 Blunt-TOPO vector using the DNA topoisomerase I enzyme, according to the protocol provided with the Zero Blunt™ TOPO™ PCR Cloning Kit for Sequencing (Thermo Fisher Scientific). Both inserts were verified by sequencing to confirm their correct amplification and antisense orientation toward the T7 promoter. The full-length *PhCNX1* and *PhCNX2* cDNA sequences were submitted to GenBank with the following accession numbers: ON052710.1 and OQ693858.1.

The plasmid bearing the full-length *PhCNX1* or *PhCNX2* cDNA was digested with Xho I or Xag I, then purified using the High Pure Product Purification Kit (Roche) following the manufacturer's protocol. A 1-μg aliquot of the linearized DNA served as a template to generate DIG-labeled molecular probes complementary to the 3’ UTR of each gene. Labeling was performed using the DIG RNA Labeling Kit (SP6/T7) according to the manufacturer's instructions (Roche).

### Transcript detection and gene expression analysis of PhCNX1 and PhCNX2 by semi-quantitative reverse transcription PCR (sqRT-PCR) and real-time quantitative reverse transcription PCR (qRT-PCR) methods

To detect *PhCNX1* and *PhCNX2* transcripts in *Petunia* anthers at different developmental stages, sqRT-PCR experiments were performed following the protocol by Lenartowski et al. [35]. In brief, a 1-µl aliquot of first-strand cDNA served as a template for PCR amplification with Platinum™ II Taq Hot-Start DNA Polymerase (Thermo Fisher Scientific) and gene-specific primers (Table 1). These primers were designed based on the *PhCNX1/PhCNX2* cDNA sequences or the *18S rRNA* EST sequence from *Petunia.* PCR cycles were carried out as follows: 95 °C for 90 s, then 33 cycles of 95 °C for 30 s, 60 °C for 30 s, and 72 °C for 30 s, with a final extension step of 72 °C for 4 min. Amplified DNA fragments were separated and visualized on a 2% agarose gel in 1 × TAE buffer. Fluorescence signals of the PCR products were captured using the ChemiDoc™ Touch Imaging System (Bio-Rad) and subsequently analyzed with Image Lab 6.1 software (Bio-Rad).

Expression of *PhCNX1* (ON052710.1) and *PhCNX2* (OQ693858.1) in developing anthers was analyzed by qRT-PCR. To ensure accurate quantification, we normalized expression to the reference *18S rRNA* gene, selected from five candidates (*18S rRNA* AJ236020.1; *actin 11* SGN-U208507; *ribosomal protein S13* SGN-U208260; *glyceraldehyde-3-phosphate dehydrogenase* SGN-U209515 and *tubulin beta-6 chain* SGN-U207876) based on the stable expression in sqRT-PCR (data not shown). The qRT-PCR primers for *PhCNX1* and *PhCNX2* were designed from non-conserved regions of the genes using Primer3Plus software.

PCR reactions (10 µl) contained 1-μl cDNA template, 0.25 μl each primer for the gene to a final concentration of 250 nM (Table 1), 7 μl nuclease-free water, and 2 μl HOT FIREPol EvaGreen® qPCR Mix Plus (no ROX) (Solis BioDyne OÜ). Reactions were run on a LightCycler®96 (Roche), using the following thermal profile: 95 °C for 12 min, then 60 cycles of 95 °C for 15 s, 60 °C for 20 s, and 72 °C for 20 s. SYBR Green fluorescence was recorded after each elongation step. Specificity of the amplifications was checked by melting curve analysis performed by heating the samples from 65 to 97 °C with temperature increments of 0.2 °C/s, with simultaneous fluorescence detection (5 readings/°C). Non-RT controls verified the absence of genomic DNA. The threshold cycle (*Ct*) was calculated based on the Second Derivative Maximum method using the LightCycler®96 Instrument Software (Roche). All reactions were repeated three times with biological replicates, and non-template controls were included.

To determine the PCR efficiencies, standard curves were generated for both the target and control genes using a series of cDNA dilutions as templates. The qRT-PCR data were plotted as fluorescence signal versus cycle number. PCR efficiencies were calculated from the slopes of the standard curves, using the equation: E = 10^[−1/slope]^. The efficiency values and *R*^*2*^ coefficient values of the qPCR standard curves are listed in Table S1. The relative level of gene expression was calculated by the Pfaffl method [36]. The normalized transcript levels of each *PhCNX1* or *PhCNX2* gene in dyads/tetrads, microspores, and pollen grains were expressed as fold change relative to the calibrator sample representing microsporocytes.

### Stain-free western blot analysis and specificity of the primary anti-CNX1/2 antibody

Immunoblotting was carried out as previously [30], with some modifications. Briefly, anthers at different developmental stages were homogenized in liquid nitrogen and extracted in a buffer containing 50 mM HEPES (pH 7.5), 10% sucrose, 5 mM EGTA, 2 mM DTT and cOmplete™ Protease Inhibitor Cocktail (Roche). Homogenates were centrifuged, and soluble proteins concentration was measured spectrophotometrically using DC™ Protein Assay (Bio-Rad) on an Infinite 200 PRO® reader (Tecan). Equal amounts of proteins were denatured at 60 °C for 15 min, centrifuged, and separated on a 10% TGX stain-free gel (Bio-Rad), prepared according to the manufacturer’s instructions and run at 140 V for 90 min. After electrophoresis, stain-free gels were activated using the ChemiDoc™ Touch Imaging System (Bio-Rad), and proteins were semi-dry transferred to an Immune-Blot LF PVDF Membrane (Bio-Rad). Fluorescence of trihalo-modified proteins present on the membrane was captured on the ChemiDoc™ Touch Imaging System (Bio-Rad). Blots were then probed with a commercial anti-CNX1/2 antibody (Agrisera), washed, and incubated with a horseradish peroxidase-conjugated secondary antibody (Merck). Signals were detected using the Amersham ECL Advance Western Blotting Detection Kit (Cytiva) on the ChemiDoc™ Touch Imaging System (Bio-Rad). Chemiluminescent band intensities were normalized to the total post-transfer protein (acquired earlier using ImageLab™ software version 6.0.1; Bio-Rad) and further normalized to a calibrator sample representing microsporocytes. All experiments were repeated three times using biological replicates.

To verify the specificity of the primary anti-CNX1/2 antibodies (Agrisera), immunoblotting was performed using *Petunia* anther proteins and *Arabidopsis* proteins as the positive control.

### Fluorescent in situ hybridization (FISH) and immunocytochemistry in *Petunia* anthers

FISH of *PhCNX1* and *PhCNX2* transcripts in *Petunia* anthers was performed according to the protocol described previously [32], with slight modifications. Selected anthers were fixed in freshly prepared 4% (*v*/*v*) formaldehyde (Polysciences) and 0.25% (*v*/*v*) glutaraldehyde in phosphate-buffered saline (PBS, pH 7.2) for 1 h at room temperature, with slight vacuum infiltration, followed by overnight fixation at 4 °C. The fixed samples were washed in PBS (pH 7.2), dehydrated through a graded series of ethanol, embedded in LR Gold resin (SPI Supplies), sectioned into semi-thin sections (cross sections of the anther), and transferred onto microscope slides covered with Biobond (BB International). *PhCNX1* and *PhCNX2* transcripts were localized with the DIG-labeled antisense molecular probes (generated above) used at final concentrations of 0.0108 µg/µl and 0.0137 µg/µl, respectively. Prehybridization and hybridization steps were carried out in 50% formamide, 4 × SSC, 5 × Denhardt’s, 1 mM EDTA and 50 mM sodium phosphate buffer for 1 h at 42 °C and overnight at 37 °C, respectively. Next, the sections were incubated with a primary mouse anti-DIG antibody (Roche), diluted 1:100 in PBS (pH 7.2) containing 0.01% (*w*/*v*) bovine serum albumin (BSA), for 2 h at room temperature. To detect FISH signals, the sections were treated with a secondary goat anti-mouse Alexa Fluor Plus 488 antibody (Invitrogen), diluted 1:100 in PBS (pH 7.2) with 0.05% (*w*/*v*) BSA, for 1 h at room temperature. As a negative control, the molecular probes were omitted. In the final step, the sections were stained with 2µg/ml Hoechst 33342 (Thermo Fisher Scientific), mounted in ProLong™ Gold Antifade Mountant reagent (Thermo Fisher Scientific), and examined with a Nikon Eclipse 80i fluorescence microscope.

Immunocytochemical localization of CNX in *Petunia* anthers was performed as described previously [32], with slight modifications. Selected anthers were fixed and embedded as described above. Semi-thin and ultra-thin sections (cross sections of the anther) were transferred onto microscope slides covered with Biobond (BB International) or collected on Formvar-coated nickel grids, respectively. For immunofluorescence labeling, the sections were first incubated in 50 mM glycine in PBS (pH 7.2) for 10 min, followed by treatment with 2% (*w*/*v*) BSA in PBS (pH 7.2) for 20 min. After blocking, the sections were incubated with a primary rabbit anti-CNX1/2 antibody (Agrisera) diluted 1:50 in PBS (pH 7.2) with 0.5% (*w*/*v*) BSA for 2 h, and then with a secondary goat anti-rabbit Alexa Fluor Plus 594 antibody (Invitrogen) diluted 1:100 in PBS (pH 7.2) with 0.5% (*w*/*v*) BSA for 1 h. For immunogold labeling, the sections were first incubated for 10 min in 50 mM glycine in PBS (pH 7.2), followed by a 15 min blocking step with 3% (*v*/*v*) BSA-c™ (Aurion) in PBS (pH 7.2). The sections were then treated with the anti-CNX1/2 primary antibody (Agrisera) diluted 1:75 in PBS (pH 7.2) with 0.3% (*v*/*v*) BSA-c™ (Aurion) for 2 h, and then with a goat anti-rabbit IgG 20 nm gold-conjugated secondary antibody (BB International), diluted 1:100 in PBS (pH 7.2) with 0.3% (*v*/*v*) BSA-c™ (Aurion) for 1 h. All the steps were conducted at room temperature. A negative control was performed by omitting the primary antibody, and the specificity of the antibody in *Petunia* tissues was confirmed by immunoblotting. Finally, the semi-thin sections were incubated with 2µg/ml Hoechst 33342 (Thermo Fisher Scientific), mounted in ProLong™ Gold Antifade Mountant reagent (Thermo Fisher Scientific), and analyzed using a Nikon Eclipse 80i fluorescence microscope. The ultra-thin sections were post-stained with 2.5% uranyl acetate and examined with a Jeol EM 1010 transmission electron microscope.

### FISH and immunocytochemistry in *Petunia* pollen and pollen tubes

FISH of *PhCNX1* and *PhCNX2* mRNA and immunocytochemical localization of CNX in cultivated pollen and pollen tubes of *Petunia* were performed as described previously [31, 33], with slight modifications. In brief, for FISH the samples were fixed in freshly prepared 4% (*v*/*v*) formaldehyde (Polysciences) in PBS (pH 7.2) and then enzymatically digested using a mixture of 1% (*w*/*v*) cellulase R10 (Serva) and 27 U of pectinase (Merck) per mL in 0.01 M citrate buffer (pH 4.8) for 25 min at 37 °C. Next, the cells were permeabilized with 0.1% (*w*/*v*) saponin in PBS (pH 7.2), followed by 0.1% (*v*/*v*) Triton X-100 (Sigma) in PBS (pH 7.2). *PhCNX1* and *PhCNX2* transcripts were localized with the DIG-labeled molecular probes (generated above) at final concentrations of 0.0129 µg/µl and 0.0149 µg/µl, respectively. Pre-hybridization and hybridization steps were performed at 42 °C, in a solution containing 50% formamide, 4 × SSC, 5 × Denhardt’s solution, 1 mM EDTA, and 50 mM sodium phosphate buffer (pH 7.0), for 30 min and overnight, respectively. Subsequently, the samples were treated with a primary mouse anti-DIG antibody (Roche) diluted 1:100 with PBS (pH 7.2), for 1 h at room temperature followed by overnight at 4 °C. The hybridization signals were detected by using a secondary anti-mouse Alexa Fluor 488 antibody (Invitrogen) diluted 1:100 in PBS (pH 7.2), for 2 h at room temperature. Negative controls were processed in the same way except that no probe was added. In the final step, DNA was stained with 2µg/ml Hoechst 33342 (Thermo Fisher Scientific) and the samples were placed onto microscope slides and covered with ProLong™ Gold Antifade Mountant reagent (Thermo Fisher Scientific). FISH images were acquired using Olympus FLUOVIEW software package connected to FLUOVIEW FV3000 confocal microscope.

For the immunofluorescent localization of CNX, the samples were fixed, enzymatically digested and permeabilized as described above, except for the permeabilization step with Triton X-100. After blocking with 1% (*w*/*v*) BSA in PBS (pH 7.2) for 10 min, the cells were incubated with a primary rabbit anti-CNX1/2 antibody (Agrisera) at a dilution of 1:100 in PBS (pH 7.2) containing 0.01% (*w*/*v*) BSA. This incubation was carried out for 1 h at room temperature, followed by overnight incubation at 4 °C. To detect signals, the samples were treated with a secondary goat anti-rabbit Alexa Fluor Plus 594 antibody (Invitrogen) prepared in a solution of 0.01% (*w*/*v*) BSA in PBS (pH 7.2), for 2 h at room temperature. Control experiments were conducted by omitting the primary antibody. In the final step, DNA was stained with 2µg/ml Hoechst 33342 (Thermo Fisher Scientific). The samples were covered with ProLong™ Gold Antifade Mountant reagent (Thermo Fisher Scientific), and images were acquired using Olympus FLUOVIEW software package connected to FLUOVIEW FV3000 confocal microscope.

### Sequence selection and comparative analyses

The nucleotide and predicted amino acid sequences used in the analyses were obtained from commonly available databases: NCBI (http://www.ncbi.nlm.nih.gov), KEGG (http://www.genome.jp/kegg), Sol Genomics Network [37], and Phytozome (https://phytozome-next.jgi.doe.gov). Firstly, sequences were filtered based on the databases’ annotations, taxonomic classification, and BLAST alignment (https://blast.ncbi.nlm.nih.gov/Blast.cgi) to verify the *CNX1* sequence from *Arabidopsis thaliana*. Next, we assessed the presence of an open reading frame and both 5’ and 3’ (UTRs in the distinguished *CNX* cDNAs). Predicted amino acid sequences obtained from the selected cDNAs began with a methionine residue at the N-terminus and ended with a motif similar to the ER-retention signal (RKPRRE or with conservative replacement).

Structural motif analyses of the *PhCNXs* were performed based on a comparative assay using data published in the literature and software adapted for predicting structural motifs, such as MotifScan [38] and InterPro [39]. The multiple alignment of predicted amino acid *Ph*CNX isoforms and sequences from selected plant species was performed by using Clustal Omega software [40]. Predicted amino acid sequences were obtained using Compute pI/Mw [41].

## Results

### *PhCNXs* cloning and sequences data

**Fig. 2:**
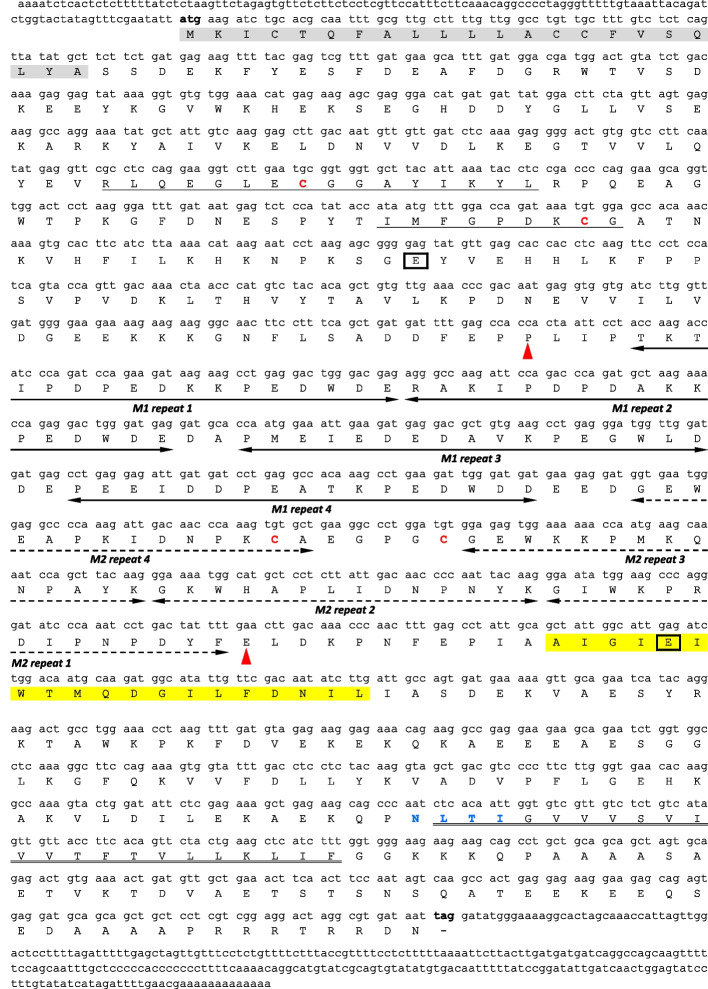
Nucleotide sequence of *PhCNX1* cDNA and its predicted amino acid sequence. The predicted signal peptide is marked in grey. CRT-family signature motifs calreticulin 1 and 2 are underlined by a single line. The double underline indicates transmembrane region of the protein. The quadruple repeats M1 (PXXIXDP(E/D)(A/D)XKP(E/D)DWD(D/E)) and M2 (GXWXXPXIXNPXYX) are shown by two-headed solid and dotted arrows, respectively. The sequence enclosed by the two red arrowheads includes the proline-rich domain. The region corresponding to the amphipathic helix is highlighted in yellow. The cysteine residues involved in disulfide bonds formation and potential N-glycosylation sites are marked in red and blue fonts, respectively. The strictly conserved glutamic acid residues are boxed. The start and termination codons are indicated in bold

**Fig. 3:**
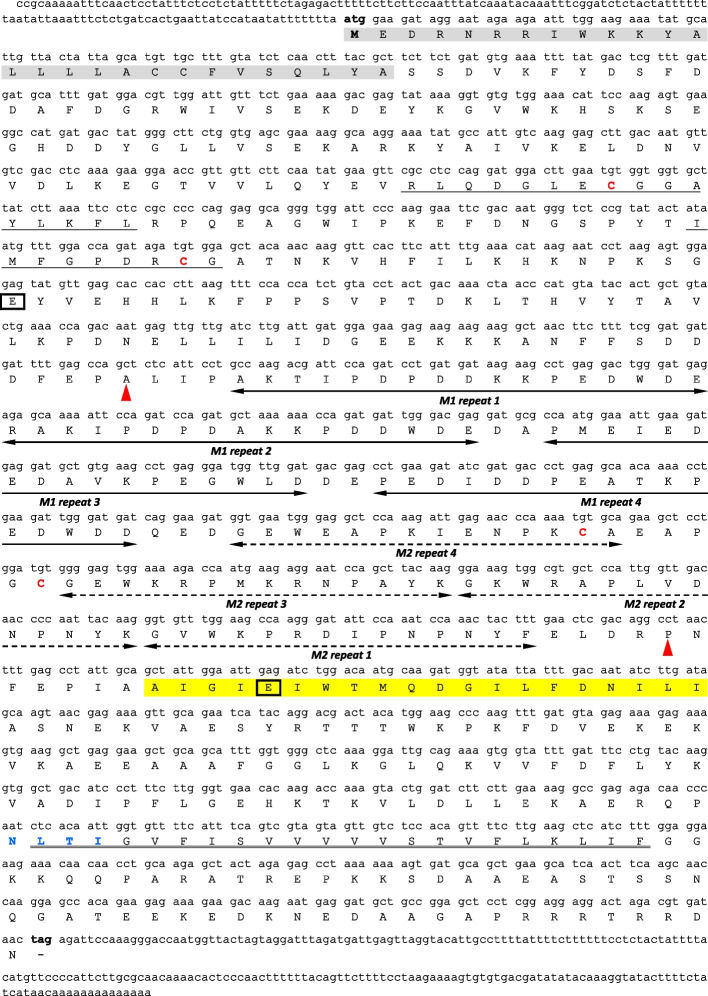
cDNA and predicted amino acid sequences of *PhCNX2*. The predicted signal peptide is highlighted in grey. The single solid lines indicate CRT-family signature motifs calreticulin 1 and 2. The transmembrane region is double underlined. The quadruple repeats M1 (PXXIXDP(E/D)(A/D)XKP(E/D)DWD(D/E)) and M2 (GXWXXPXIXNPXYX) are marked with two-headed solid and dotted arrows, respectively. The region contained between red arrowheads indicates the proline-rich domain. The amphipathic helix is highlighted in yellow. The cysteine residues responsible for formation of disulfide bonds are shown in red font, while blue font indicates the potential N-glycosylation site. The strictly conserved glutamic acid residues are boxed. The start and stop codons are marked in bold

The full-length *Arabidopsis thaliana* cDNA sequences, NM_125573.4 (described as *AtCNX1*) and NM_120816.3 (described as *AtCNX2*), were used to search the Sol Genomics Network database. Two *Petunia axilliaris* cDNA sequences were identified: Peaxi162Scf00314g00733.1 and Peaxi162Scf00207g00250.1. As both sequences lacked complete 5’ or 3’ untranslated regions (UTRs), gene-specific primers were designed to clone the missing ends via rapid amplification of cDNA ends. The resulting 5’ or 3’ fragments were sequenced and used to design a set of primers to amplify the full-length *PhCNX1* and *PhCNX2* cDNAs, which were subsequently cloned into the pCR4 Blunt-TOPO vector. The obtained *PhCNX1* (ON052710.1) and *PhCNX2* (OQ693858.1) sequences showed the highest identity scores of 68.73% and 67.82%, respectively, with *AtCNX1* and *AtCNX2*. The *PhCNX1* cDNA sequence is 1,996 bp in length and consists of a 121-bp 5’ UTR upstream of the ATG initiation codon, a 1,605 bp open reading frame (ORF) terminating with a TAG stop codon, and a 270-bp 3’ UTR (Fig. 2). The *PhCNX2* cDNA sequence is 1,975 bp in length and consists of a 144-bp 5’ UTR upstream of the ATG initiation codon, a 1,620 bp ORF terminating with a TAG stop codon, and a 211-bp 3’ UTR (Fig. 3). Comparison of the two coding sequences revealed that they are highly homologous, sharing 84.6% nucleotide sequence identity. No putative polyadenylation signal sequences, such as ATTAAA or TTAAAT, as described by Nevins [42], were mapped at the 3’-end of either clone. However, non-canonical motifs, such as YAYTG or CAYTG (Y = C or T), which may potentially play a role in this process, according to Joshi [43], were found at positions 1939 bp and 1819 bp in the *PhCNX1* and *PhCNX*2 clones, respectively.

### Analysis of predicted amino acid sequences of PhCNX1 and PhCNX2 clones

The putative *PhCNX1* and *PhCNX2* ORFs encode polypeptides of 534 and 539 amino acids, respectively. Both proteins are highly conserved, showing 85.6% sequence identity. Compute pI/Mw software indicates a theoretical molecular weight of 60.4 kDa for *Ph*CNX1 (WAK43332.1) and 61.5 kDa for *Ph*CNX2 (XBC19607.1), as well as slightly acidic peptides having isoelectric points of 4.74 and 4.95, respectively. Structural-motif detection revealed the characteristic domain organization for CNX/CRT family members (Figs. 2 and 3): a globular N-domain starting with a predicted signal peptide, a central proline-rich P-domain, and a short C-domain. Two conserved CNX/CRT family signature motifs, calreticulin 1 and 2, each containing a cysteine residue responsible for disulfide-bridge formation, were identified. Other typical P domain elements are the M1 (PXXIXDP(E/D)(A/D)XKP(E/D)DWD(D/E)) and M2 (GXWXPPXIXNPXYX) tandem repeats, which occur four times each [7, 44–46] and form an asymmetric hairpin structure [44]. Additionally, the M1 repeats include Ca^2+^-binding motifs [47], while the second pair of cysteine residues in M2 repeats can form disulfide-bridge. A transmembrane region and an amphipathic α-helix sequence, relevant for ER retention, were also predicted [7]. Two strongly conserved lectin-binding sites formed by acidic glutamic acid residues and clustered around the glucose-binding site were mapped [44]. The NetPhos3.1 [48] predicted numerous potential serine, threonine, and tyrosine phosphorylation sites in both *Ph*CNX1 and *Ph*CNX2, mainly in their N- and C-domains. Moreover, a single *N*-glycosylation site was identified within each of the analyzed polypeptides using an *N*-glycosylation consensus sequence NXS/T (X ≠ N or P) [49].

Predicted *Ph*CNX sequences were compared to 14 verified CNX isoforms derived from various plant species (Fig. S1). The sequences were selected based on database descriptions, as well as experimental verification. Despite discrepancies observed at the N- and C-termini, alignment revealed a high overall identity. *Ph*CNX1 shares 68–75% identity with other CNX1 sequences, whereas *Ph*CNX2 shares 72–74% identity with the analyzed CNX2 sequences.

### Expression pattern of PhCNX1 and PhCNX2 genes during pollen development

**Fig. 4:**
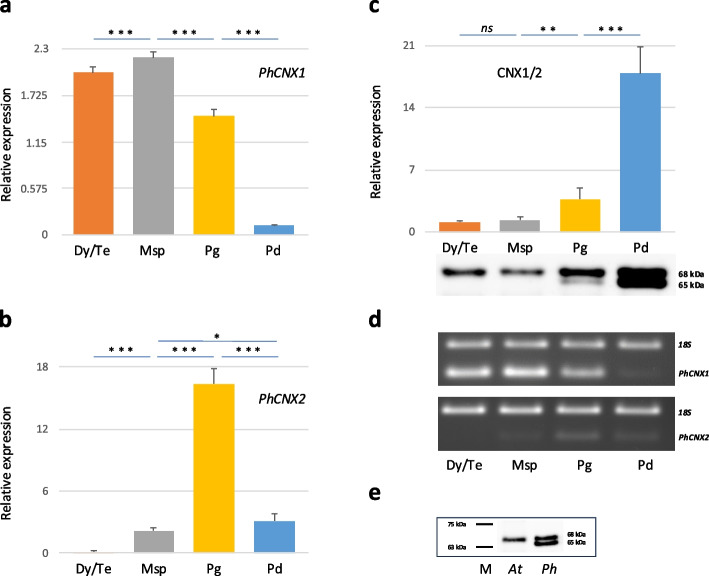
qRT-PCR analysis of *PhCNX1* (**a**) and *PhCNX2* (**b**) mRNA levels, and stain-free western blot of CNX (**c**), followed by sqRT-PCR analysis of *PhCNX1/2* levels (**d**) in whole *Petunia* anthers during successive stages of pollen development (*Msc* microsporocyte, *Dy*/*Te* dyad/tetrad, *Msp* microspore, *Pg* pollen grain stages) and in dry pollen (*Pd*), and specificity control of the anti-CNX1/2 primary antibody (**e**). *Graphs* in** a** and **b** show relative *PhCNX1* and *PhCNX2* mRNA levels (mean ± SD of three biological and two technical replicates) normalized to *Ph18S* rRNA levels. **c** *Graph* shows relative CNX levels (mean ± SD of eight biological replicates) normalized to total protein post-transfer; a cropped representative western blot is shown below (full blots in Fig. S2a and b). **d** sqRT-PCR detection of *PhCNX1* and *PhCNX2* transcripts in whole *Petunia* anthers; *Ph18S* was co-amplified as reference. Cropped representative agarose gels are shown below (full gels in Fig. S3a and b). **e** Specificity of the CNX1/2 antibody was confirmed by immunoblotting with crude protein extracts from whole *Arabidopsis* (*At* line 2) and *Petunia* dry pollen (*Ph* line 3); original blot in Fig. S4. Statistical analysis for qRT-PCR and stain-free western blot experiments was carried out by one-way ANOVA (*ns,* not significant; *p ≤ 0.05; **p ≤ 0.01; ***p ≤ 0.001). Transcript and protein levels are expressed as fold change relative to the *Msc* stage calibrator

To analyze the expression patterns of *PhCNX* genes, we first examined transcript levels of *PhCNX1* and *PhCNX2* at successive stages of pollen development, including microsporocyte, dyad/tetrad, microspore, and pollen grain stages, as well as in dry pollen collected from mature anthers at dehiscence. Using qRT-PCR analysis, we observed a progressive increase in *PhCNX1* gene expression from microsporocytes through dyad/tetrad stage to microspores (Fig. 4a). The highest *PhCNX1* mRNA level occurred after meiosis completion, at the microspore stage, coinciding with sporoderm formation, when both microspores and tapetal cells exhibit extremely high secretory activity correlated with protein synthesis and folding in the ER. Subsequently, the level of *PhCNX1* transcripts declined during the pollen grain phase, reaching a minimum in dry pollen (Fig. 4a). We next analyzed *PhCNX2* mRNA levels by qRT-PCR at the same successive stages of pollen development (Fig. 4b). Our results demonstrated a drop in *PhCNX2* mRNA levels between the microsporocyte and dyad/tetrad stages, followed by a steady increase through the microspore stage, and culminating at the pollen grain stage, where *PhCNX2* mRNA levels were about sevenfold higher than at the microspore stage. Subsequently, a marked decline in *PhCNX2* mRNA levels was detected in dry pollen. All changes in *PhCNX1* and *PhCNX2* expression levels across the selected developmental stages of pollen development were statistically significant.

Next, the western blot analysis of total proteins isolated from the whole *Petunia* anthers and dry pollen using an antibody against *Arabidopsis thaliana* CNX1/2 was performed (Fig. 4c). At the microsporocyte, dyad/tetrad, and microspore stages, we detected relatively low levels of CNX. However, once the male gametophyte formed, we observed a statistically significant, almost twofold increase in CNX level at the pollen grain stage compared to microspore stage, followed by a peak in dry pollen, where protein levels were fivefold higher than at the pollen grain stage. The high CNX level in dry pollen may indicate its accumulation, potentially involved in the maturation processes of newly synthesized peptides required for the upcoming germination and pollen tube growth. Interestingly, only a single band of around 68 kDa was observed before and during meiosis. After meiosis, at the pollen grain stage, as well as in dry pollen, two bands of 65 and 68 kDa were detected (Fig. 4c). These findings indicate a possible post-translational modification of the polypeptide or the presence of two CNX proteins at these developmental stages of *Petunia* pollen. A similar pattern of immunoblotting was obtained in the primary antibody specificity control (Fig. 4e). In the soluble protein fractions isolated from *Petunia* dry pollen, the antibody against CNX1/2 recognized two bands of approximately 65 kDa and 68 kDa (Fig. 4e, *lane 3*). In contrast, the positive control—whole *Arabidopsis* plants—showed only one band at approximately 66 kDa (Fig. 4e, *lane 2*). For comparison, sqRT-PCR results for *PhCNX1* and *PhCNX2* transcript levels are presented below the protein-levels chart (Fig. 4d).

### Spatiotemporal distribution of PhCNX1 and PhCNX2 transcripts in developing anther

**Fig. 5:**
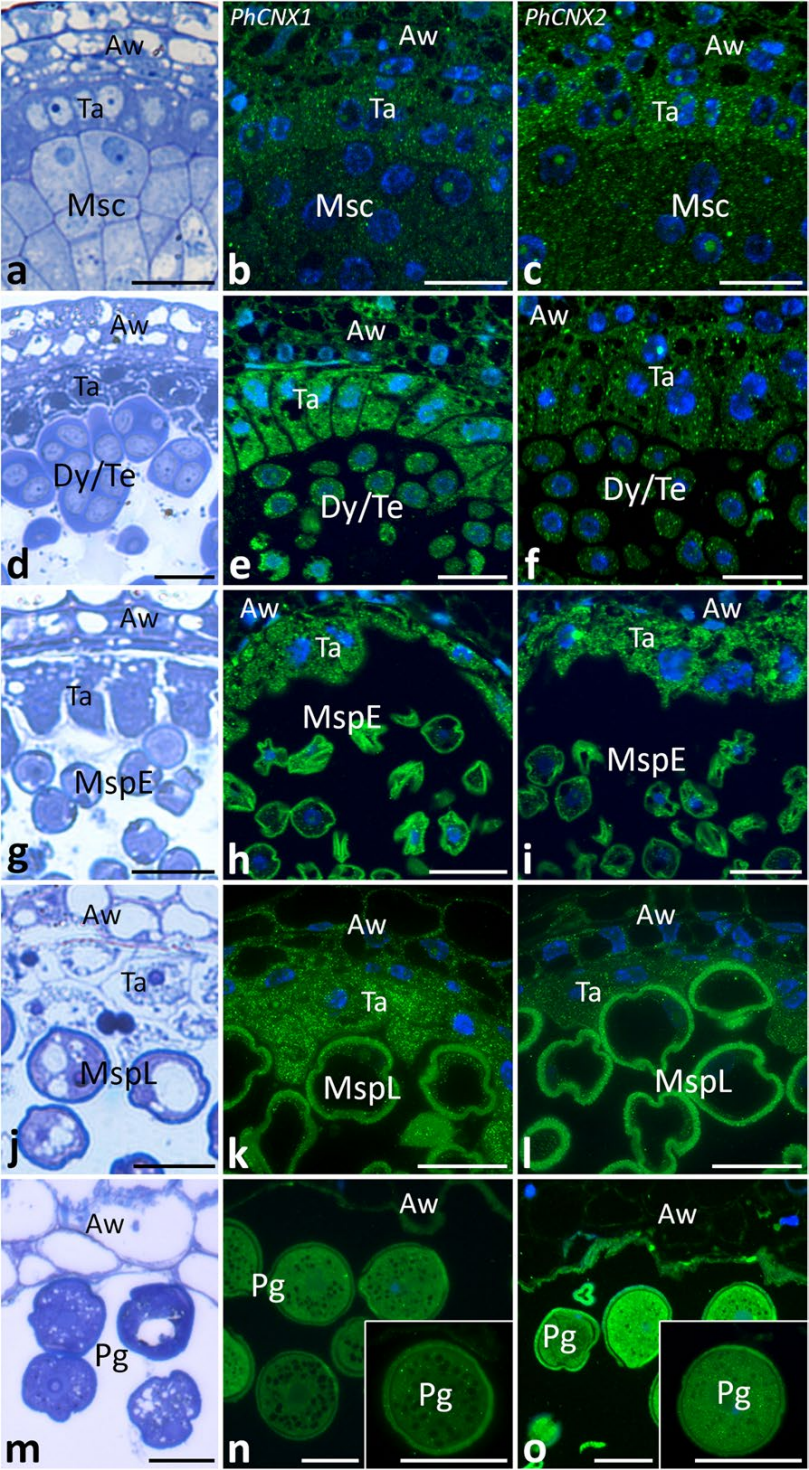
Localization of *PhCNX1* (*green, ***b**, **e**, **h**, **k**, **n**) and *PhCNX2* (*green,* **c**, **f**, **i**, **l**, **o**) transcripts using FISH during subsequent stages of pollen development (*Msc* microsporocyte, *Dy*/*Te* dyad/tetrad, *MspE* early microspore, *MspL* late microspore, *Pg* pollen grain stages). The *insets* with white outlines on **n** and **o** show magnifications of the pollen grains. Methylene blue staining (**a**, **d**, **g**, **j**, **m**). Nuclei are stained with Hoechst 33342 (*blue*, **b**, **c**,** e**, **f**, **h**, **i**,** k**, **l**, **n**, **o**). *Aw* anther wall, *Ta* tapetum. *Scale bars* correspond to 20 µm

To examine the spatiotemporal distribution of *PhCNX1* and *PhCNX2* transcripts in developing pollen and somatic cells of *Petunia* anthers, we used the FISH technique. Initially, semi-thin cross sections of the anthers were stained with methylene blue to identify various developmental stages, including microsporocytes (Fig. 5a), dyads/tetrads (Fig. 5d), early microspores (Fig. 5g), late microspores (Fig. 5j), and maturing pollen grains (Fig. 5m). Before meiosis, *PhCNX1* mRNAs were distributed throughout the cytoplasm of microsporocytes, tapetal cells, and cells comprising the anther wall (Fig. 5b). A similar transcripts localization pattern was observed during meiosis, when dyads and tetrads are formed, with the strongest hybridization signals occurring in the cytoplasm of tapetal cells (Fig. 5e). After the completion of meiosis, at the early and late microspore stages, *PhCNX1* mRNA remained in the germline and somatic cells of the anther (Fig. 5h, k), showing weak FISH signals in the microspore cytoplasm, but strong green fluorescence in tapetal cells. This result is consistent with our qRT-PCR analysis, which shows a peak of *PhCNX1* expression at the microspore stage (Fig. 4a), correlated with the extremely high metabolic activity of the tapetum. In two-celled pollen grains, FISH signal decreased and *PhCNX1* mRNAs were detected mainly in pollen grain cytoplasm (Fig. 5n). Microscopic analysis of *PhCNX2* transcripts distribution also confirmed their presence during pollen development in *Petunia* anther. Before and during meiotic division, *PhCNX2* mRNAs were detected in the cytoplasm of both microsporocytes and meiocytes, as well as in somatic cells of the anther. However, at these developmental stages, the highest FISH signals were observed in the tapetal cells (Fig. 5c, f). After meiosis, when microspores were released from the callosic cell wall surrounding the tetrads, *PhCNX2* mRNAs were still present in the germline and somatic cells of the anther. The hybridization signals were detected predominantly in the cytoplasm of the tapetal cells, whereas both early and late microspores and anther wall cells showed weaker labeling (Fig. 5i, l). The most significant increase in FISH signal was observed at the maturing pollen stage, when *PhCNX2* transcripts accumulated mainly in the cytoplasm of pollen grains. At this stage, very little FISH signal was detected in the somatic cells of the anther (Fig. 5o). No labeling was observed in the no-probe control (data not shown).

### Spatiotemporal distribution of CNX proteins during microsporogenesis

**Fig. 6:**
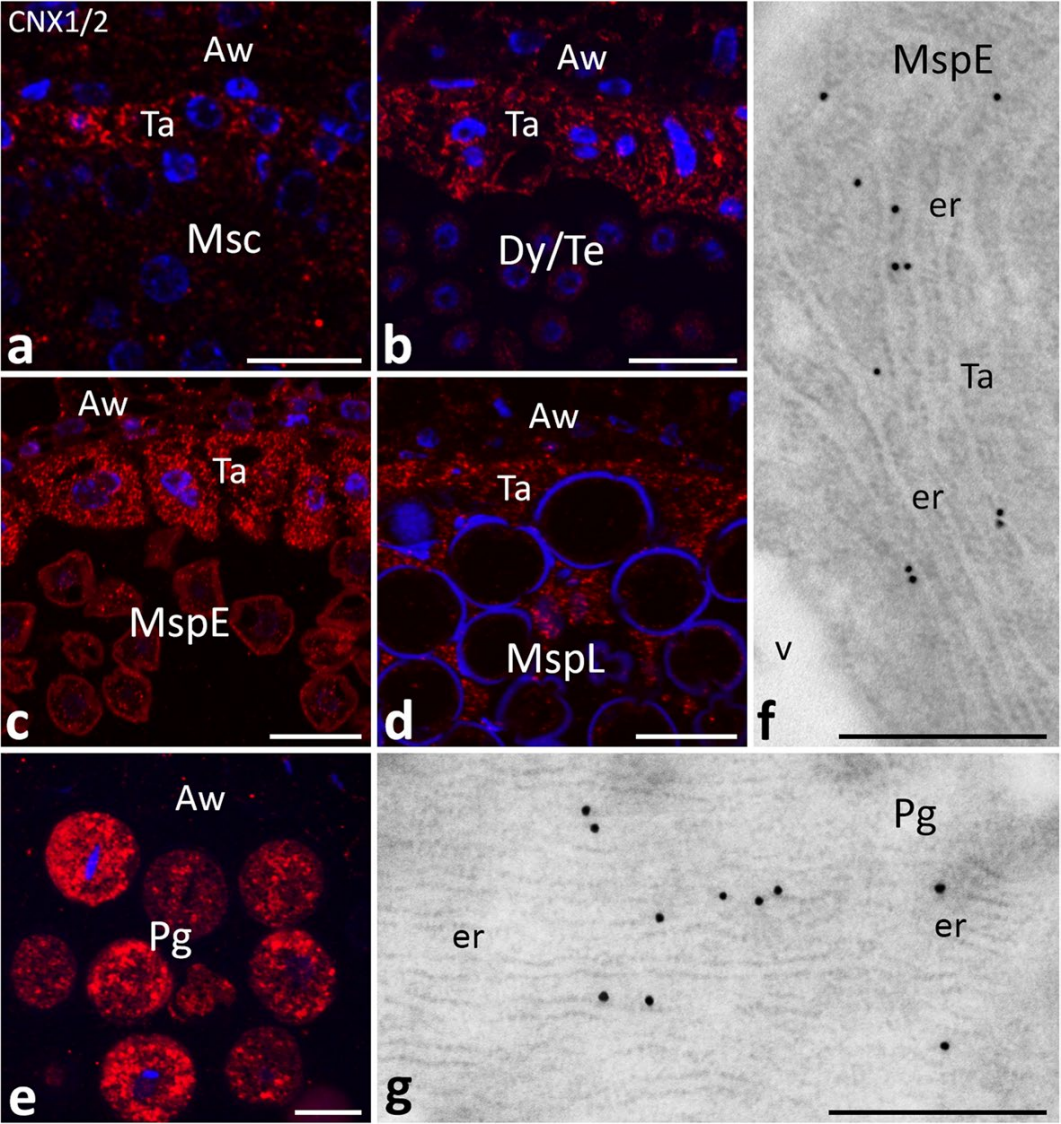
Localization of CNX protein using immunofluorescence (*red*, **a**-**e**) and immunogold (**f**, **g**) techniques during subsequent stages of pollen development (*Msc* microsporocyte, *Dy*/*Te* dyad/tetrad, *MspE* early microspore, *MspL* late microspore, *Pg* pollen grain stages). Nuclei are stained with Hoechst 33342 (*blue*, **a**-**e**). *Aw* anther wall, *er* endoplasmic reticulum, *Ta* tapetum, *v* vacuole. *Scale bars* correspond to 20 µm (**a**-**e**), and 500 nm (**f**, **g**)

To further investigate CNX distribution within pollen chambers at different stages of pollen development, we used the immunofluorescence technique. Semi-thin sections stained with the primary anti-CNX1/2 and a fluorochrome-conjugated secondary antibody revealed the protein in both germline and somatic anther cells, with signal intensity varying by defined developmental stages and cell types. Prior to meiosis, the labeling appeared in microsporocytes, as well as in somatic cells, with the tapetal cells exhibiting the highest signal intensity (Fig. 6a). During meiosis, as dyads and subsequently tetrads were formed, CNX was still present in the cytoplasm of the germline and tapetal cells, again with a stronger signal observed in the latter (Fig. 6b). A similar pattern of CNX localization was also observed after meiosis, with predominant immunolabeling detected in the tapetal cells, while early and late microspores displayed a weaker immunofluorescence signal (Fig. 6c, d). As the mature pollen developed, a significant increase in CNX was observed in the cytoplasm of pollen grains, with no labeling in the residual tapetum or in anther wall cells (Fig. 6e). This result is consistent with our western blot analysis and confirms that the highest accumulation of CNX in developing anther correlates with the pollen grain stage (Fig. 4c). Because CNX is an ER transmembrane protein, we tested its ER localization in *Petunia* anthers via the immunogold technique (Fig. 6f, g). Indeed, electron microscopy of ultrathin sections through the anthers confirmed several gold traces (corresponding to the CNX labeling) in the ER of both the tapetal cells (Fig. 6f) and pollen grains (Fig. 6g).

### Distribution of PhCNX1 and PhCNX2 transcripts and CNX during pollen germination and pollen tube growth

**Fig. 7:**
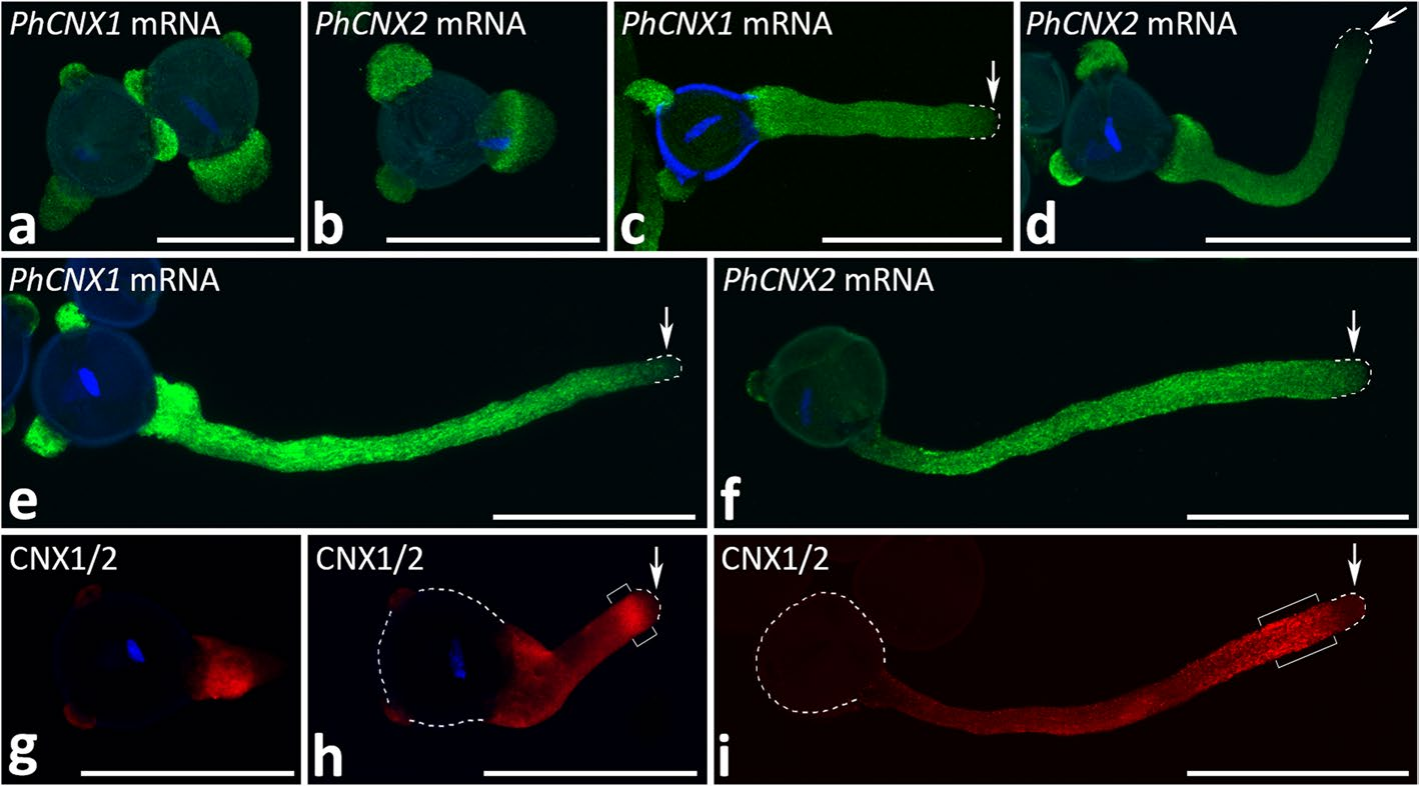
Localization of *PhCNX1* (*green,* **a**, **c**, **e**) and *PhCNX2* (*green,* **b**, **d**, **f**) transcripts using FISH, and distribution of CNX protein (*red*, **g**-**i**) using immunofluorescence in *Petunia* germinating pollen (**a**, **b**, **g**), short (**c**, **d**, **h**), and elongated (**e**, **f**, **i**) pollen tubes. *Arrows* in **c**-**f**,** h** and** i** show low/lack of the fluorescence in the clear zone of short and elongated pollen tubes. *Bracketed regions* in **h** and **i** show CNX accumulation in the cytoplasm of the subapical zone of the pollen tubes. *Dashed lines* in **c**-**f**, **h** and **i** indicate the outlines of pollen grains or pollen tube tips. Nuclei are stained with Hoechst 33342 (*blue*, **a**-**h**). *Scale bars* correspond to 25 µm (**a**), and 50 µm (**b**-**i**)

To determine the spatial distribution of *PhCNX1* and *PhCNX2* mRNAs and CNX proteins in cultivated pollen/pollen tubes, we performed FISH and immunostaining experiments using confocal microscopy. In germinating pollen grains, both *PhCNX1* and *PhCNX2* transcripts were predominantly detected in the apertures, including the germinal aperture where the tube emerges and non-active ones (Fig. 7a, b). A similar pattern of hybridization was also preserved during pollen tube growth (Fig. 7c-f). In the cytoplasm of the elongating pollen tubes, both *PhCNX1* and *PhCNX2* transcripts were equally distributed from the base of the tube to the sub-apex (Fig. 7c-f), but not to the apical zone (Fig. 7c-f, arrows), which correlates with the presence of rough ER (rER) in elongating pollen tubes (Fig. 1h; [31, 50]). The localization pattern of CNX protein differed slightly from the distribution pattern of the tested transcripts, since in both germinating pollen and cultivated pollen tubes, the main site of CNX detection was the germinal aperture (Fig. 7g), and then the growing pollen tubes (Fig. 7h, i). In both short and elongated tubes, the protein was distributed throughout the cytoplasm of the tubes, with a prominent accumulation in the subapical zone (Fig. 7h, i, bracketed regions) and a very weak signal in the apical cytoplasm (Fig. 7h, i, arrows). It should be noted that the subapical zone is a site of particular smooth ER (sER) accumulation in growing pollen tubes (Fig. 1h; [50]).

## Discussion

In this study, we report the first comprehensive cloning and characterization of two *CNX* homologs in *Petunia hybrida*, *PhCNX1* and *PhCNX2*, and investigate their expression patterns and localization during microsporogenesis, pollen germination, and pollen tube growth. Bioinformatic analyses revealed that *Ph*CNX1 and *Ph*CNX2 isoforms are highly conserved across species yet exhibit notable species-specific differences, potentially reflecting functional specialization. Their high sequence identity with CNX homologs in other plants highlights the evolutionary importance of these proteins in maintaining ER protein folding and quality control across plant species [25, 26]. Notably, despite the existence of multiple CNX isoforms in plants, certain regions of their sequences are highly conserved. Our in silico analysis of the predicted *Ph*CNX amino acid sequences identified domains characteristic of the CNX/CRT family [45, 46], suggesting that *Ph*CNX1 and *Ph*CNX2 function as ER molecular chaperones, similar to their homologs in other species.

Given the high structural conservation of *Ph*CNX1 and *Ph*CNX2 and the principal role of CNX in the ER, we analyzed their expression profiles in the context of their probable roles in pollen development, pollen germination and pollen tube growth. While the importance of chaperones, including CNX, in pollen development has been established through *Arabidopsis* mutant studies [29, 51], the expression dynamics of *CNX* genes during successive stages of microsporogenesis remain unexplored. This is particularly significant because male gametophyte development in angiosperms involves a complex, tightly coordinated interplay between gametophytic and sporophytic tissues, mediated by precisely regulated gene expression patterns. Transcriptomic studies indicate that pollen formation is controlled by two subsequent developmental programs—early and late—each associated with stage-specific gene expression patterns [52, 53]. Early genes are activated soon after meiosis in microspore and bicellular pollen, and their expression declines as the pollen matures. In contrast, late genes are expressed after asymmetric mitosis and their mRNAs accumulate in mature pollen [54, 55]. Many late—phase genes show pollen-specific expression patterns, and function in signaling, vesicle trafficking, cell wall metabolism, membrane transport, and cytoskeleton dynamics during pollen germination and pollen tube growth [52, 56]. Our qRT-PCR analysis revealed that in developing anther, *PhCNX1* and *PhCNX2* exhibit differential expression profiles, which may reflect their association with these developmental programs. *PhCNX1* showed peak expression during the microspore stage, suggesting its potential classification as an early-phase gene, with its transcriptional activity precisely coinciding with the period of intense sporoderm synthesis and deposition. In contrast, *PhCNX2* expression peaked at the mature pollen stage, suggesting association with the late developmental program and possible roles in pollen maturation and germination preparedness. Strikingly, both *PhCNX* transcripts were markedly reduced in dehydrated pollen, mirroring *Arabidopsis* transcriptomic data showing downregulation of chaperone mRNAs, including CNX1/2, in mature/dry pollen [57]. The decline in *PhCNX* transcript levels may reflect the transition of the dry pollen into a metabolically “quiescent” state with suppressed transcriptional activity. Interestingly, western blot analysis demonstrated a progressive increase in CNX protein levels during another development, followed by its significant accumulation in dry pollen, where translation is known to be silenced. This observation is consistent with previous reports indicating that mature/dry pollen accumulates both mRNAs and proteins [57–61], including molecular chaperones like CNX as confirmed by proteomic analyses [57].

To complement our expression analysis, we performed microscopic localization studies. In somatic anther tissues, CNX and its transcripts were predominantly localized in the cytoplasm of tapetal cells, from microsporocyte to microspore stages. These results likely reflect the high metabolic activity of the tapetum, consistent with reports indicating particularly tight regulation of *CNX* expression in plant tissues with high energy demands [24, 25]. *Petunia* has the most common type of tapetum (secretory type), which exhibits features of metabolically active cells, including the presence of numerous, well-developed ER cisternae [62]. This tissue secretes nutrients, proteins, lipids, and enzymes for microsporocytes and developing microspores, while simultaneously initiating programmed cell death to provide critical materials for pollen wall formation during the later stages of their development. Given the intensive protein synthesis required for these processes, tapetal CNX likely ensures proper folding and quality control of secretory proteins. CNX chaperone activity could be equally important in *Petunia* anther germline cells, where *PhCNX* mRNAs were mainly detected during the post-meiotic period, coinciding with the processes of intense sporoderm synthesis in the developing pollen. Interestingly, microscopic observations revealed substantial CNX accumulation in the cytoplasm of *Petunia* pollen grains, correlating with western blot results. This cytoplasmic CNX pool may participate in critical processes such as protein folding and quality control, protection against abiotic stress, and supporting pollen recovery during rehydratation and subsequent pollen tube growth in pistil tissues.

Pollen tube elongation, a process regulated by tightly coordinated gene expression [63], exhibited distinct *PhCNX* mRNA and CNX localization patterns. During early germination, the transcripts initially accumulated in pollen apertures, while in the elongating tube cytoplasm, *CNX* mRNAs were uniformly distributed (excluding the clear zone), contrasting with protein accumulation in the subapical region. Similar results were previously obtained for CRT and its transcripts [31, 34]. These findings indicate that: (1) these proteins are translated on ribosomes present in the pollen tube cytoplasm from its base to the subapical zone, an area enriched in rER [34]; and (2) both proteins accumulate particularly in the subapical zone of the growing pollen tube [31, 34] and current report, corresponding to a region rich in sER cisternae [50]. It should be noted that our previous ultrastructural analyses of *Petunia* pollen tubes confirmed this pattern of the rER/sER distribution, consistent with observations in other angiosperm species [31, 34]. As a molecular chaperone in the ER, CNX may play an important role in cellular processes associated with the rapid tip growth of pollen tubes. Our previous studies have shown that pollen tube elongation depends on CRT, which may participate in maintaining Ca^2^⁺ homeostasis, protein synthesis, and glycoprotein quality control [31, 33]. The role of CNX/CRT cycle proteins in pollen tube growth was also confirmed by Vu et al. [29], who showed that *Arabidopsis cnx1 crt1 crt2 crt3* mutants exhibit impaired pollen germination and tube elongation, demonstrating the essential function of CNX/CRT cycle proteins in these processes. Given that the extremely rapid growth of pollen tubes requires very high rates of protein synthesis, we propose that, similar to the anther, CNX chaperone may be involved in the quality control of newly synthesized peptides. This hypothesis, however, requires further studies.

## Conclusions

Our results demonstrate that *PhCNX*s are dynamically expressed throughout pollen development in the anther and during subsequent pollen germination and tube elongation. The observed spatiotemporal regulation of *PhCNX* expression strongly suggests its function as a molecular chaperone supports the intensive protein biosynthesis required for successful male gametophyte development and the polarized growth of the pollen tube.

## Supplementary Information

Supplementary Material 1: Table S1. Parameters of standard curves.

Supplementary Material 2: Fig. S1. Amino acid sequence alignment of CNX 1 and CNX2 isoforms derived from selected plant species. The multiple alignment of *Ph*CNX1 (WAK43332.1) and *Ph*CNX2 (XBC19607.1) proteins with predicted amino acid sequences from miscellaneous plant species, including *Arabidopsis thaliana* (NM_125573.4, NM_120816.3); *Arabidopsis lyrata* (XM_021024190.1, XM_021020166.1); *Capsella rubella* (XM_023786065.1, XM_023780637.1); *Tradescantia hirsutiflora* (KU530113.1); *Glycine max* (AB196933.1); *Pisum sativum* (Y17329.1); *Eutrema salsugineum* (XM_006399154.2); *Camelina sativa* (XM_010424918.1); *Zea mays* (NM_001156845.1, NM_001308610.1); *Oryza sativa* (XM_015779952.2). The numbers on the right show amino acid position. Asterisks and yellow backlight indicate fully conserved amino acid residues, while colons and dots show conservation between groups of strongly (> 0.5 in the Gonnet PAM 250 matrix) and weakly (≤ 0.5 in the Gonnet PAM 250 matrix) similar properties, respectively.

Supplementary Material 3: Fig. S2. An uncropped stain-free blot with total protein post-transfer (**a**) from whole *Petunia* anthers during subsequent stages of pollen development (*Msc* microsporocyte, *Dy*/*Te* dyad/tetrad, *Msp* microspore, *Pg* pollen grain stages) and dry pollen (*Pd*), and immunoblot (**b**). The blot edges are marked with *solid lines.*

Supplementary Material 4: Fig. S3. An uncropped agarose gels showing sqRT-PCR products resoled on 2% agarose gels in 1 × TAE. *Ph18S and PhCNX1* (**a** line 2–6) and *Ph18S* and *PhCXN2* (**b** line 2–6) at different stages of pollen development: *Msc* microsporocyte, *Dy*/*Te* dyad/tetrad, *Msp* microspore, *Pg* pollen grain stages, and *Pd* dry pollen; *K* reaction without template DNA; *M* DNA ladder (Perfect Plus 1 kb DNA Ladder, EURx).

Supplementary Material 5: Fig. S4. An uncropped immunoblot with total protein extracts from the whole plant of *Arabidopsis* (*At* line 1) and *Petunia* dry pollen (*Ph* line 2). The *arrows* indicate the location of the CNX protein(s). The blot edges are marked with *solid lines*. *M* protein marker (Protein Marker VI, Applichem).

## Data Availability

The datasets used and/or analyzed during the current study are available from the corresponding author on reasonable request.

## Abbreviations

Ca^2+^: Calcium ions
*CNX/CNX*: Calnexin gene/protein
CRT: Calreticulin
ER: Endoplasmic reticulum
rER: Rough endoplasmic reticulum
sER: Smooth endoplasmic reticulum
