# Supplementary material for: Molecular cloning and the expression profile of two calnexin genes – CNX1 and CNX2 – during pollen development and pollen tube growth in Petunia
Source: BMC Plant Biol. 2025 Oct 23;25:1449. doi: 10.1186/s12870-025-07186-2 (PMC12548239; doi:10.1186/s12870-025-07186-2)
Supplement: Supplementary file 1 — Supplementary Material 1: Table S1. Parameters of standard curves. [file 12870_2025_7186_MOESM1_ESM.pdf]

gene	efficiency	R^2
18S rRNA	1.90	1
PhCNX1	1.78	0.99
PhCNX2	1.95	0.98
