# Supplementary material for: Molecular cloning and the expression profile of two calnexin genes – CNX1 and CNX2 – during pollen development and pollen tube growth in Petunia
Source: BMC Plant Biol. 2025 Oct 23;25:1449. doi: 10.1186/s12870-025-07186-2 (PMC12548239; doi:10.1186/s12870-025-07186-2)
Supplement: Supplementary file 2 — Supplementary Material 2: Fig. S1. Amino acid sequence alignment of CNX 1 and CNX2 isoforms derived from selected plant species. The multiple alignment of PhCNX1 (WAK43332.1) and PhCNX2 (XBC19607.1) proteins with predicted amino acid sequences from miscellaneous plant species, including Arabidopsis thaliana (NM_125573.4, NM_120816.3); Arabidopsis lyrata (XM_021024190.1, XM_021020166.1); Capsella rubella (XM_023786065.1, XM_023780637.1); Tradescantia hirsutiflora (KU530113.1); Glycine max (AB196933.1); Pisum sativum (Y17329.1); Eutrema salsugineum (XM_006399154.2); Camelina sativa (XM_010424918.1); Zea mays (NM_001156845.1, NM_001308610.1); Oryza sativa (XM_015779952.2). The numbers on the right show amino acid position. Asterisks and yellow backlight indicate fully conserved amino acid residues, while colons and dots show conservation between groups of strongly (> 0.5 in the Gonnet PAM 250 matrix) and weakly (≤ 0.5 in the Gonnet PAM 250 matrix) similar properties, respectively. [file 12870_2025_7186_MOESM2_ESM.pdf]

```
A.thaliana_CNX1       --MRQRQLFSVF---L---LLLAFV----SFQKLCYCDDQTVLYESFDEPFDGRWIVSKNSDYEGVWKHAKSEGHEDYGLLVSEKARKYGIVKEL--DEPLNLKEGTVVLQYEV   100
A.lyrata_CNX1         --MRQRQLFSGF---L---LLLAFV----SFQKLCYCDDQTVLYESFDEPFDGRWIVSKNSDYEGVWKHAKSEGHEDYGLLVSEKARKYGIVKEL--DEPLNLKEGTVVLQYEV   100
C.rubella_CNX1        --MRERQLFSVF---L---LLLAFV----SFQKLCYCDDQTVLYESFDEPFDGRWIVSKNGEYEGVWKHAKSEGHDDYGLLVSEKARKYGIVKEL--DEPLNLKEGTVVLQYEV   100
T.hirsutiflora_CNX1   -----MTIAPKI---L---LLCLLS----SLW-LQIWASEPIFYESFDEDFEGRWIKSEKEGYSGVWKHAKSDGHDDYGLLVSEKARKYAIVKKL--DEPISLKDETIVLQFEV   96
P.hybrida_CNX1        -----MKICTQF---A---LLLLAC----CFVSQLYASSDEKFYESFDEAFDGRWTVSDKEEYKGVWKHEKSEGHDDYGLLVSEKARKYAIVKEL--DNVVDLKEGTVVLQYEV   97
G.max_CNX1            --MGERKGIPMALGLLAMILFFIASS-SSHLVRASGDADDAIFYESFDEDFDGRWIVSDKEDYNGVWKHAKSDGHDDYGLLVSEQARKYAIVKEL--AESVSLKDGTVVLQFET   109
P.sativum_CNX1        --MVDRKEIPLAMGLLAVLLFFVASSSSFHLVRASDEVDDAIFYESFDEDFDNRWIVSGKEEYNGVWKHSKSEGHDDFGLLVSEPARKYAIVKEL--DAPVSLKDGTVVLQFET   110
Z.mays_CNX1           ------MGGRAL---L---LLLLVS----ALV-FQIHASDPLLYEPFDEDFEGRWVVSKKDEYQGVWRHAKSDGHEDYGLLVSEKARKYAIIKEL--DEPVTLKDGTVVLQFEV   95
O.sativa_CNX1         -----MVGGRAL---L---PLLLLS----ALL-VQIRASDPLFYEPFDESFEGRWVLSGKDDYKGVWKHEKSDGHEDYGLLVSEKAKKYAIIKEL--DEPVTLKDGTVVLQFEV   96
A.thaliana_CNX2       --MRERIITFVS---L---LLVALL----SFPSVSYCDDQTILYESFDEPFDGRWVVSEKAEYQGVWKHEKSEGHDDYGLLVSEKAKKYGIVKELDVDEPLNLNEGTVVLQYEA   102
A.lyrata_CNX2         --MRERILTFVS---L---LLVALL----SFSNLCYCDDQTILYESFDEPFHGRWIVSEKSEYQGVWKHEKSEGHDDYGLLVSEKAKKYGIVKELDVDEPLNLKEGTVVLQYEA   102
C.rubella_CNX2        --MRERTLTFVS---L---LLLALV----SFSNLCHCDDQTVLYESFEEPFDGRWIVSEKAEYQGVWKHEKSEGHDDHGLLVSEKAKKYGIVKEL--DEPLNLKEGTVVLQYEA   100
P.hybrida_CNX2        MEDRNRRIWKKY---A---LLLLAC----CFVSQLYASSDVKFYDSFDDAFDGRWIVSEKDEYKGVWKHSKSEGHDDYGLLVSEKARKYAIVKEL--DNVVDLKEGTVVLQYEV   102
E.salsugineum_CNX2    --MRERTLTFVS---V---LLVALV----SFQSLCYCDEQTILYESFDEPFDGRWIVSEKPEYQGAWKHEKSQGHDDYGLLVSEKARKYGIVKEL--DEPLNLKEGTIVLQYEA   100
C.sativa_CNX2         --MRERVSTFVS---L---LLVALF----SFSSLCYCDDQTVLYESFDESFDGRWIVSEKPEYQGVWKHEKSEGHDDYGLLVSEKAKKYGIVKEL--DEPLNLKDGTVVLQYEA   100
Z.mays_CNX2           -----MMGGRAL-------LLLLVS----ALL-VQIRASDPLLYEPFDEDFEGRWIVSKKDEYQGVWKHAKSDGHEDYGLLVSDKARKYAIIKEL--DEPVTLNDGTVVLQFEV   95
                                          :         :       .:  :*: *:: *..**  * :  *.*.*:* **:**:*.*****: *:**.*:*:*     : *:: *:***:*.

A.thaliana_CNX1       RFQEGLECGGAYLKYLRPQEAGWTPQGFDSESPYSIMFGPDKCGGTNKVHFILKHKNPKSGEYVEHHLKFPPSVPYDKLSHVYTAILKPDNEVRILVDGEEKKKANLLSGEDFE   214
A.lyrata_CNX1         RFQEGLECGGAYLKYLRPQEAGWTPQGFDSESPYSIMFGPDKCGATNKVHFILKHKNPKSGEYVEHHLKFPPSVPYDKLSHVYTAILKPDNEVRILVDGEEKKKANLLSGEDFE   214
C.rubella_CNX1        RFQEGLECGGAYLKYLRPQEAGWTPQGFDSESPYSIMFGPDKCGATNKVHFILKHKNPKSGEYVEHHLKFPPSVPYDKLSHVYTAILKPDNEVRILVDGEEKKKANLLSGEDFE   214
T.hirsutiflora_CNX1   RLQEGLECGGAYLKYLRPQDAGWTPKGFDNESPYSIMFGPDKCGSTNKVHFILNHKNPKSGKYVEHHLKYPPSVPSDKLSHVYTAILKPDNELRILIDGEEKKKANFLSSDDFE   210
P.hybrida_CNX1        RLQEGLECGGAYIKYLRPQEAGWTPKGFDNESPYTIMFGPDKCGATNKVHFILKHKNPKSGEYVEHHLKFPPSVPVDKLTHVYTAVLKPDNEVVILVDGEEKKKGNFLSADDFE   211
G.max_CNX1            RLQNGLECGGAYIKYLRPQESGWKPKEFDNESPYSIMFGPDKCGATNKVHFIFKHKNPKSGEYVEHHLKYPPSVPSDKLTHVYTAILKPDNELQILIDGEEKKKANFLSSEDFE   223
P.sativum_CNX1        RLQNGLECGGAYIKYLQTQESGWKPKGFDNESGYSIMFGPDRCGATNKVHFIFRHKNPKTGKHVEHHLKFPPSVPSDKLSHVYTAVLKDDNEVSILIDGEEKKKANFLSSEDFE   224
Z.mays_CNX1           RLQNGLECGGAYIKYIRPQDAGWDAKEFDNETPYTIMFGPDKCGSTNKVHFILKHKNPKTGKYVEHHLKFPPSVPYDKLSHVYTAILKPDNEVKILVDGEEKKKANFLSADDFE   209
O.sativa_CNX1         RLQNGLECGGAYLKYIRPQDAAWDAKEFDNETPYTIMFGPDKCGSTNKVHFILKHKNPKTGKYVEHHLKFPPSVPYDKLSHVYTAILKPDNEVRILVDGEEKKKANFLSADDFE   210
A.thaliana_CNX2       RFQEGLECGGAYLKYLRPQEAGWVPQGFDNDSPYSIMFGPDKCGATNKVHFILKHKNPKSGEFVEHHLKFPPSVPFDMLSHVYTAVLKSDNEVRILVDGEEKKKGNLLSAEDFE   216
A.lyrata_CNX2         RFQQGLECGGAYLKYLRPQEAGWVPQGFDNDSPYSIMFGPDKCGATNKVHFILKHKNPKSGEFVEHHLKFPPSVPFDKLSHVYTAILKPDNEVRILVDGEEKKKGNLLSAEDFE   216
C.rubella_CNX2        RFQQGLECGGAYLKYLRPQEAGWVPQGFDNDSPYSIMFGPDKCGATNKVHFILKHKNPKSGEFVEHHLKFPPSVPFDKLSHVYTAILKPDNEVRILVDGEEKKKGNLMSAEDFE   214
P.hybrida_CNX2        RLQDGLECGGAYLKFLRPQEAGWIPKEFDNGSPYTIMFGPDRCGATNKVHFILKHKNPKSGEYVEHHLKFPPSVPTDKLTHVYTAVLKPDNELLILIDGEEKKKANFFSDDDFE   216
E.salsugineum_CNX2    RFQEGLECGGAYIKYLRPQEAGWVPKGFDNESPYSIMFGPDKCGATNKVHFILKHKNPKSGEYVEHHLKFPPSVPFDKLSHVYTAILKPDNEVRILVDGEEKKKGNLLSAEDFE   214
C.sativa_CNX2         RFQEGLECGGAYLKYLRPQEAGWVPQGFDNDSPYSVMFGPDKCGATNKVHFILKHKNPKSGEFVEHHLKFPPSVPFDKLSHVYTAILKPDNEVTILVDGEEKKKGNLLSGEDFE   214
Z.mays_CNX2           RLQNGLECGGAYIKYIRPQDAGWDAKEFDNETPYTIMFGPDKCGSTNKVHFILKHKNPKTGKYVEHHLKFPPSVPYDKLSHVYTAILKPDNEVRILIDGEEKKKANFLSADDFE   209
                      *:*:********:*::: *::.*  : **. : *::*****:**.*******:.*****:*:.******:***** * *:*****:** ***: **:*******.*::* :***

A.thaliana_CNX1       PALIPAKTIPDPEDKKPEDWDERAKIPDPNAVKPEDWDEDAPMEIEDEEAEKPEGWLDDEPEEVDDPEATKPEDWDDEEDGMWEAPKIDNPKCEAAPGCGEWKRPMKRNPAYKG   328
A.lyrata_CNX1         PALIPAKTIPDPEDKKPEDWDERAKIPDPNAVKPDDWDEDAPMEIEDEEAEKPEGWLDDEPEEVDDPEATKPEDWDDEEDGMWEAPKIDNPKCEAAPGCGEWKRPMKRNPAYKG   328
C.rubella_CNX1        PALIPAKTIPDPEDKKPEDWDERAKIPDPNAVKPDDWDEDAPMEIEDEEAEKPEGWLDDEPEEVDDPEATKPEDWDDEEDGMWEAPKIDNPKCEAAPGCGEWKRPMKRNPAYKG   328
T.hirsutiflora_CNX1   PALIPLKTIPDPDDKKPADWDERPKIADSSAVKPDDWDEDAPMEIEDEEAVKPEGWLDDEPEDIDDPEATVPEDWDVEEDGEWESPKIDNPKCEVAPGCGEWKRPMKKNPAYKG   324
P.hybrida_CNX1        PPLIPTKTIPDPEDKKPEDWDERAKIPDPDAKKPEDWDEDAPMEIEDEDAVKPEGWLDDEPEEIDDPEATKPEDWDDEEDGEWEAPKIDNPKCAEGPGCGEWKKPMKQNPAYKG   325
G.max_CNX1            PPLIPSKTIPDPDDKKPEDWDERAKIPDPSAVKPDDWDEDAPMEILDEEAENPEGWLDDEPEEIDDPEATKPEDWDDEEDGEWEAPKIENPKCEAAPGCGEWKRPTKRNPAYKG   337
P.sativum_CNX1        PALIPSKTIPDPDDKKPEDWDERAKIPDPEAVKPEDWDEDAPREIIDEEAEKPEPWLDHEPEV-DDP-EAKPEDWDDEEDGEWEAPKIENPKCEAAPGCGEWKRPTKSNPAYKG   336
Z.mays_CNX1           PALIPSKTIPDPDDKKPEDWDERAKIPDPDAVKPDDWDEDAPMEIVDDEATKPEGWLDDEPEEIDDPEAAKPEDWDDEEDGEWEAPKIDNPKCEEAPGCGEWKRPMKQNPAYKG   323
O.sativa_CNX1         PSLIPSKTIPDPDDKKPEDWDERAKIPDPDAVKPDDWDEDAPMEIEDEEATKPEGWLDDEPEEIDDPEANKPEDWDDEEDGEWEAPKIDNPKCEEAPGCGEWKRPMKQNPAYKG   324
A.thaliana_CNX2       PPLIPSKTIPDPEDKKPEDWDERAKIPDPNAVKPDDWDEDAPMEIEDEEAEKPEGWLDDEPVEVEDPEASKPEDWDDEEDGEWEAPKVSNTKCEAAPGCGEWKRPMKRNPAYKG   330
A.lyrata_CNX2         PPLIPSKTIPDPEDKKPEDWDERAKIPDPNAVKPDDWDEDAPMEIEDEEAEKPVGWLDDEPEEVEDPEASKPEDWDDEEDGEWEAPKVSNPKCEVAPGCGEWKRPMKRNPAYKG   330
C.rubella_CNX2        PPLIPSKTIPDPEDKKPEDWDERAKIPDPSAVKPDDWDEEAPMEIEDEEVEKPEGWLDDEPEEVEDPEASKPEDWDDEEDGEWEAPKVSNPKCEAAPGCGEWKRPMKRNPAYKG   328
P.hybrida_CNX2        PALIPAKTIPDPDDKKPEDWDERAKIPDPDAKKPDDWDEDAPMEIEDEDAVKPEGWLDDEPEDIDDPEATKPEDWDDQEDGEWEAPKIENPKCAEAPGCGEWKRPMKRNPAYKG   330
E.salsugineum_CNX2    PPLIPSKTIPDPEDEKPEDWDERAKIPDPNAVKPDDWDEDAPMEIEDEEAEKPEGWLDDEPEEVEDPEASKPEDWDDEEDGEWEAPKVSNPKCEAAPGCGEWKRPMKRNPAYKG   328
C.sativa_CNX2         PPLIPSKTIPDPDDKKPEDWDERAKIPDPTAVKPDDWDEDAPMEIEDEEAEKPQGWLDDEPEEVDDPEASKPEDWDDEEDGEWEAPKVSNPKCETAPGCGEWKRPMKRNPAYKG   328
Z.mays_CNX2           PALIPSKTIPDPDDKKPEDWDERAKIPDPDAVKPDDWDEDAPMEIVDEEATKPEGWLDDEPEEIDDPEAAKPEDWDDEEDGEWEAPKIDNPKCEEAPGCGEWKRPMKQNPAYKG   323
                      * *** ******:*:** ***** ** *  * **:****:** ** *::. :*  ***.**   :**    ***** :*** **:**:.* **  .*******:* * ******

A.thaliana_CNX1       KWSSPLIDNPAYKGIWKPRDIPNPDYFELDRPDYEPIAAIGIEIWTMQDGILFDNILIAKDEKVAETYRQTTWKPKFDVEKEKQKAEEEA--AGSADGLKSYQKVVFDLLNKVA   440
A.lyrata_CNX1         KWSSPLIDNPAYKGIWKPKDIPNPDYFELDRPDYEPIAAIGIEIWTMQDGILFDNILIAKDEKVAETYRQTTWKPKFDVEKEKQKAEEEA--AGSADGLKSYQKVVFDLLNKVA   440
C.rubella_CNX1        KWSSPLIDNPAYKGIWKPRDIPNPDYFELDRPDYEPIAAIGIEIWTMQDGILFDNILIAKDEKVAETYRQTTWKPKFDVEKEKQKAEEEA--AGSADGLKSYQKIVFDLLNKVA   440
T.hirsutiflora_CNX1   KWHAPMIDNPDYKGIWKPQEIDNPEYFELDKPDFEPIAAIGIEIWTMQDGILFDNILIAADEKVAESYREKTWKPKYDVEKEKQKAEEAEAASSVSDGLAGFQKKVFDGLYKVA   438
P.hybrida_CNX1        KWHAPLIDNPNYKGIWKPRDIPNPDYFELDKPNFEPIAAIGIEIWTMQDGILFDNILIASDEKVAESYRKTAWKPKFDVEKEKQKAEEEA----ESGGLKGFQKVVFDLLYKVA   435
G.max_CNX1            KWSAPYIDNPSYKGIWKPREIPNPEYFELAKPDFEPIAAIGIEIWTMQDGILFDNVLIANDDKVAESYRETTWKPKFTVEKDKLKAEEEA--ATGSDGISGFQKKVFDLLYKIA   449
P.sativum_CNX1        KWSAPYIDNPNYKGIWKPQEIPNPEYFELEKPDFEPIAAIGIEIWTMQDGILFDNVLIAKDDKIAESYRETTWKPKFNIEKEKQKHEEEAA-RSESEGIAGIQKKAFDLLYKIA   449
Z.mays_CNX1           KWHAPMIDNPNYKGIWKPQEIPNPEYFELDKPDFDPIAAIGIEIWTMQDGILFDNILIADDEKVATSILEKTWKPKYDVEKEKEKAEEEA--AAGADGLSEFQKKIFDVLYKIA   435
O.sativa_CNX1         KWHAPLIDNPNYKGIWKPQEIPNPEYFELDKPDSDPIAAIGIEIWTMQDGILFDNILIADDEKVATSILEKSWKPKYEVEKEKEKAEEAA--AAAGDGLSEFQKKIFDVLYKLA   436
A.thaliana_CNX2       KWSSPLIDNPAYKGIWKPRDIPNPDYFELERPNLEPIAAIGIEIWTMQDGILFDNILISKDEKVAETYRQSTWKPKFDVEKEKQKAEDEA--AGEADGLKSYQKKVFDLLYKVA   442
A.lyrata_CNX2         KWSSPLIDNPAYKGIWKPRDIPNPDYFELERPNLEPIAAIGIEIWTMQDGILFDNILIAKDEKVAETYRQSTWKPKFDVEKEKQKAEDEA--AGEADGLKSYQKKVFDLLYKVA   442
C.rubella_CNX2        KWSSPLIDNPAYKGIWKPRDIPNPDYFELDTPNYEPIAAIGIEIWTMQDGIIFDNVLIAKDEKVAETYRQTTWKPKFDVEKEKQKAEDEA--AGETDGLKSYQKKVFDLLYKIA   440
P.hybrida_CNX2        KWRAPLVDNPNYKGVWKPRDIPNPNYFELDRPNFEPIAAIGIEIWTMQDGILFDNILIASNEKVAESYRTTTWKPKFDVEKEKVKAEEAA----AFGGLKGLQKVVFDFLYKVA   440
E.salsugineum_CNX2    KWSSPLIDNPAYKGIWKPRDIPNPDYFELDRPDFEPIAAIGIEIWTMQDGILFDNVLIAKDEKVAETYRQTTWKPKFDVEKEKQKAEDEA--AAEADGLKSYQKKVFDLLYKVA   440
C.sativa_CNX2         KWSSPLIDNPAYKGIWKPRDIPNPDYFELDRPNFEPIAAIGIEIWTMQDGIIFDNILIAKEEKVAETYRQTTWKPKSDVEKEKQKAEDEA--AGDADGLKSYQKKVFDLLYKVA   440
Z.mays_CNX2           KWHAPMIDNPNYKGIWKPQEIPNPEYFELDRPDFDPIAAIGIEIWTMQDGILFDNILIADDEKVATSILEKTWKPKYDVEKEKEKAE-EA--AAGADGLSDFQKKIFDVLYKIA   434
                      ** :* :*** ***:***::* **:****  *: :***************:***:**: ::*:* :   .:****  :**:* * *          *:   **   ** * *:*

A.thaliana_CNX1       DLSFLSAYKSKITELIEKAEQQPNLTIGVLVAIVV---VFFSLFLKLIFGGKKAAAPVEKKKPEV---------AESSKS-----G-D---EA-EKK-EETAAPRKRQPRRDN   530
A.lyrata_CNX1         DLSFLSAYKSKITELIEKAEQQPNLTIGVLVSIVV---VFFSLFLKLIFGGKKA-APVEKKKPEV---------AESSKS-----G-D---EA-EKK-EETAAPRKRQPRRDN   529
C.rubella_CNX1        DLSFLSAYKSKITELIEKAEQQPNLTVGVLVSIVV---VFFSLFVKLIFGGKKAVAPLEKKKPEV---------AESSKS-----E-D---EA-EKK-EETAAPRKRQPRRDN   530
T.hirsutiflora_CNX1   DVPFLAAYKSKIIDIIEKAEKQPNLTIGILVSVAV---VLLTILFRLFFGGRKATVVPAAPAT--NTPTEADKEADKDSGSTEEKEGNEQEENEKEEETAAQRPRRRRA----   542
P.hybrida_CNX1        DVPFLGEHKAKVLDILEKAEKQPNLTIGVVVSVIV---VTFTVLLKLIFGGKKKQPAAAASAETVK-----TDVAETSTS-----NSQATEEKEEQSEDAAAAPR-RRTRRDN   534
G.max_CNX1            DIPFLSEHKSKIFDLIEKAEKQPNLTIGILVAVVV---VFVSIFLRLIFGGKKPAKVEKKPERTEA-------SNNQGSG-----ENEENKEKEKQKEEASNAAR-RRPRRET   546
P.sativum_CNX1        DIAFLSGQKEKIIEIIEKGEKQPNLTIGIIVSVVI---VFVSIFFRLIFGGKKPANVEANVEKKKT-------NTETTSK-----QDGGEKEDNKEKEETANPPR-RRPKRDN   546
Z.mays_CNX1           DVPFLAPYKTKIIDVIEKGEKQPNITIGILVSVVV---VFVTVLFKILFGGKKPVAPV-KPAAEAKKPKATVTDGAGSSGD-------KDEKE--DEKEETAGPRRRTRRET-   534
O.sativa_CNX1         DIPFLEPYKTKIIDVIEKGEKQPNITIGILVSIVV---VFVTVLFRILFGGKKPAAPV-KPVAEVKKPKSTESEAAGSSGG-------KEDEKEEEEKDGAAAPRRRSRRET-   537
A.thaliana_CNX2       DISFLSAYKSKIMELIEKAETQPNLTIGVLISIVI---VFLSLFFKLIFGG-AKAKV-EKKKPETA--------AETSTS-----E-A---KTEEKA-EAVAAPRKRQTRRES   532
A.lyrata_CNX2         DISFLSAYKSKIMELIEKAETQPNLTIGVLISVVV---VFLSLFFKLIFGG-AKAKV-EKKKPETA--------AETSTS-----E-A---KTEEKE-ETAPVPRKRQTRRES   532
C.rubella_CNX2        DISFLSAYKSKIMELIEKAETQPNLTMGVLISVVL---VFLSIFFKLIFGG-AKVNV-EKKKPETA--------TEASTS-----E-T---KTEEKG-ETAAVPRKKQTRRDS   530
P.hybrida_CNX2        DIPFLGEHKTKVLDLLEKAERQPNLTIGVFISVVV---VVSTVFLKLIFGGKKQQPARATREPKKS-----DAAEASTSS-----NQGATEEKEDKNEDAAGAPR-RRTRRDN   539
E.salsugineum_CNX2    DISFLSAYKSKIMELIEKAETQPNLTIGVLVSVVV---VFVSLFFKLIFGGGAKVNV-EKKKPESR--------TETSTS-----E-A---KTEEKE-EAAPAPRKRQTRRES   531
C.sativa_CNX2         DISFLSAYKSKIMELIEKAETQPNLTIGMIISIVVVVLSFLSLFFKLIFGG-AKVNV-EKKKPETA--------TEASSS-----E-S---KTEEKE-ERAAVPRKRPTRRDS   533
Z.mays_CNX2           DIPFLAPYKTKIIDVIEKGEKQPNITIGILVSVVI---VFVTVLFKILFGGKKPVAPV-KPAAEAKKPKAAETDGAGSSGD-------KDEKE--DEKDE-TGPRRRTRRET-   532
                      *: **   * *: :::**.* ***:*:*:.::: :      :::.:::***                          .            :   .         * :
```
