# Supplementary figures and images for: Molecular cloning and the expression profile of two calnexin genes – CNX1 and CNX2 – during pollen development and pollen tube growth in Petunia
Source: BMC Plant Biol. 2025 Oct 23;25:1449. doi: 10.1186/s12870-025-07186-2 (PMC12548239; doi:10.1186/s12870-025-07186-2)

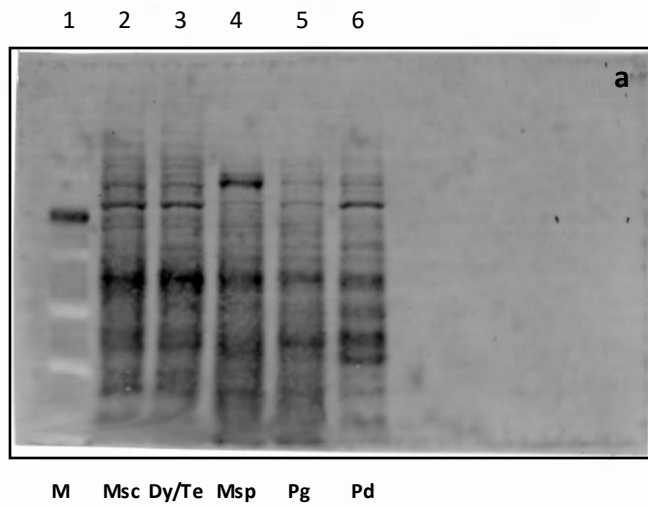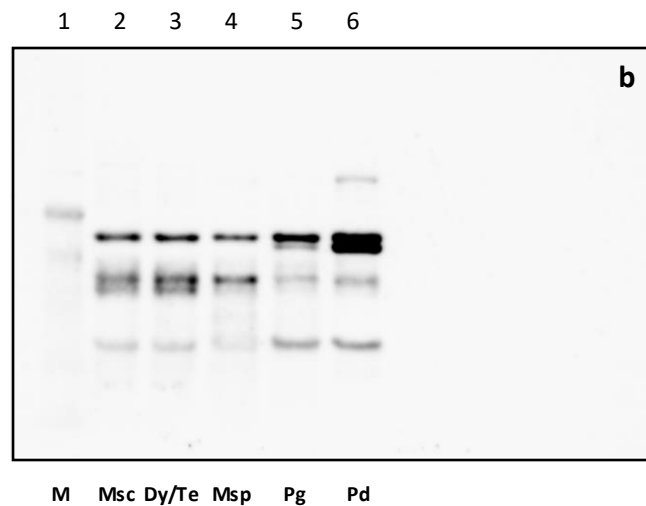

Supplement: Supplementary file 3 — Supplementary Material 3: Fig. S2. An uncropped stain-free blot with total protein post-transfer (a) from whole Petunia anthers during subsequent stages of pollen development (Msc microsporocyte, Dy/Te dyad/tetrad, Msp microspore, Pg pollen grain stages) and dry pollen (Pd), and immunoblot (b). The blot edges are marked with solid lines. [file 12870_2025_7186_MOESM3_ESM.pdf]

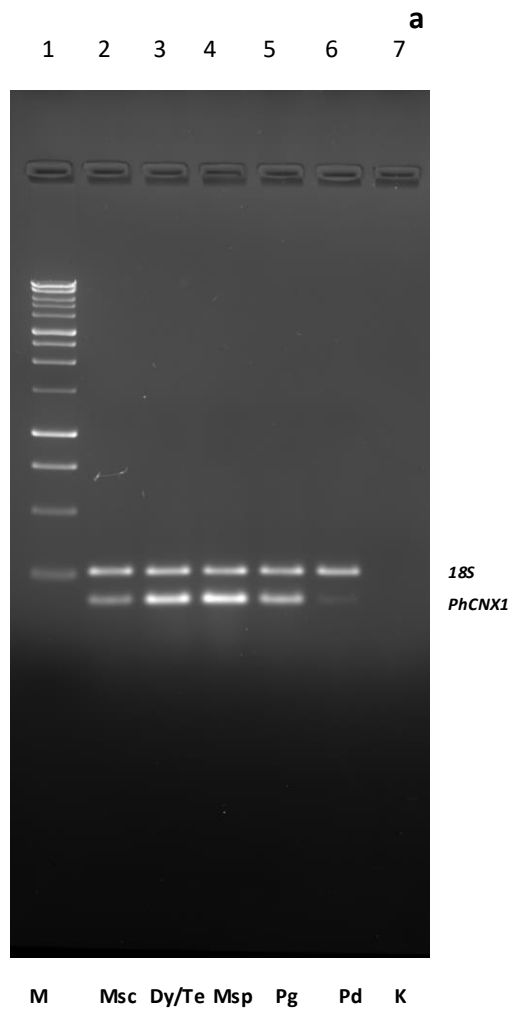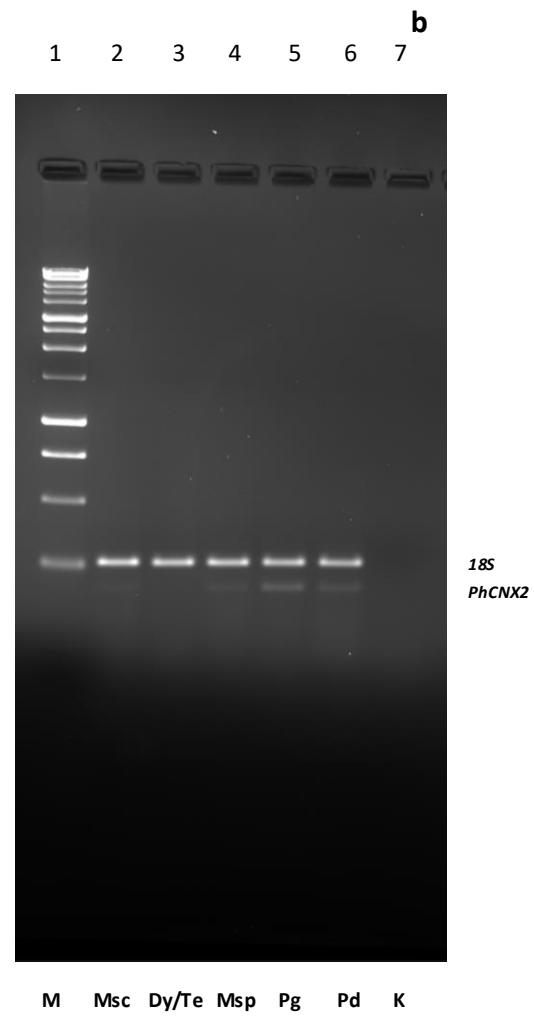

Supplement: Supplementary file 4 — Supplementary Material 4: Fig. S3. An uncropped agarose gels showing sqRT-PCR products resoled on 2% agarose gels in 1 × TAE. Ph18S and PhCNX1 (a line 2–6) and Ph18S and PhCXN2 (b line 2–6) at different stages of pollen development: Msc microsporocyte, Dy/Te dyad/tetrad, Msp microspore, Pg pollen grain stages, and Pd dry pollen; K reaction without template DNA; M DNA ladder (Perfect Plus 1 kb DNA Ladder, EURx). [file 12870_2025_7186_MOESM4_ESM.pdf]

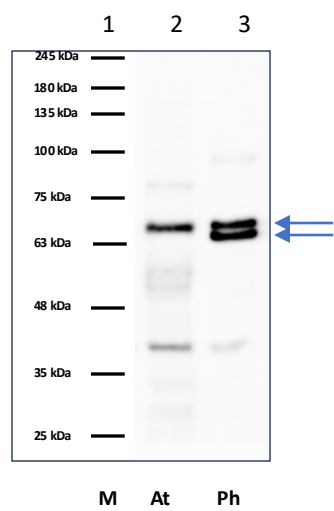

Supplement: Supplementary file 5 — Supplementary Material 5: Fig. S4. An uncropped immunoblot with total protein extracts from the whole plant of Arabidopsis (At line 1) and Petunia dry pollen (Ph line 2). The arrows indicate the location of the CNX protein(s). The blot edges are marked with solid lines. M protein marker (Protein Marker VI, Applichem). [file 12870_2025_7186_MOESM5_ESM.pdf]
